# Schizophrenia risk gene ZNF536 modulates retinoic acid response and neuronal gene networks in SH-SY5Y cells

**DOI:** 10.3389/fnmol.2025.1671354

**Published:** 2025-10-14

**Authors:** Artemiy O. Kurishev, Dmitrii A. Abashkin, Dmitry S. Karpov, Ekaterina V. Marilovtseva, Yulia A. Chaika, Ekaterina V. Semina, Vera E. Golimbet

**Affiliations:** ^1^Mental Health Research Center, Moscow, Russia; ^2^Engelhardt Institute of Molecular Biology, Russian Academy of Sciences, Moscow, Russia

**Keywords:** ZNF536, schizophrenia, SH-SY5Y, retinoic acid, neuronal differentiation, transcriptome, CRISPR/Cas9, enhancers

## Abstract

ZNF536, a brain-specific transcriptional repressor, has recently emerged as a candidate risk gene for schizophrenia (SZ), yet its functional role in human neurodevelopment remains poorly understood. We used CRISPR/Cas9 genome editing to generate a dual-allelic ZNF536 knockout model in SH-SY5Y cells, combining a 103 kb deletion encompassing SZ-associated intronic regions with a disruption of zinc finger domains in exon 2. We performed transcriptome profiling of mutant cells undergoing all-trans retinoic acid (ATRA)-induced differentiation and analyzed neurite outgrowth phenotypes. Knockout cells exhibited impaired activation of retinoic acid receptor (RAR) target genes, reduced neurite outgrowth, and failure of neuronal maturation. Gene set enrichment analysis uncovered dysregulation of E2F4-mediated cell cycle pathways. The targeted intronic deletion altered the expression of multiple SZ-associated genes, supporting the functional importance of cis-regulatory elements within ZNF536. These findings identify ZNF536 as a critical regulator of RA-responsive gene networks and neuronal differentiation, modulating neurogenic commitment through coordinated control of transcriptional repression and cell proliferation, and offer new mechanistic insights into its contribution to schizophrenia pathogenesis.

## Introduction

Schizophrenia is a debilitating psychiatric disorder with a complex genetic architecture, driven by the cumulative effect of numerous genetic variants, each conferring a modest increase in risk. This polygenic nature has historically hindered efforts to pinpoint causative genes, as traditional linkage studies lacked the power to detect these subtle contributions (Henriksen et al., 2017). However, genome-wide association studies (GWAS) have transformed this landscape, identifying over 287 genomic loci statistically associated with SZ (Trubetskoy et al., 2022), though most lie in non-coding regions, posing challenges for mechanistic interpretation (Ripke et al., 2013; Dennison et al., 2020). In 2025 cross-ancestry analyses incorporating diverse populations (96,806 cases and 492,818 controls) revealed 61 additional novel loci and prioritizing potential causal genes including ACE, CNNM2, and SNAP91 (Dang et al., 2025). Among the loci associated with SZ, genes encoding several transcription factors (TFs), including ASCL1, EGR1, FOXP1, KLF6, POU3F2, SATB2, and TCF4, have been found to control the expression of multiple genes involved in neurogenesis, neuronal projection formation, and synaptic transmission (Karpov et al., 2024).

Complementing these GWAS findings, meta-analysis of whole-exome sequencing data has identified rare variants—including copy number variations (CNVs) and protein-truncating sin-gle-nucleotide variants (SNVs) in 10 specific genes that substantially elevate SZ risk (Singh et al., 2022). One of them is SP4—a TF critical to neuronal gene expression. The overlap in evidence on transcription factors—with SP4 identified across both rare variant analyses and GWAS—highlights the way such master regulators of gene expression networks represent a mechanistic link from genetic risk through to the neurobiological phenotypes identified across SZ (Iyegbe and O'Reilly, 2022). Within this complex genetic landscape, TFs have emerged as significant risk factors for SZ pathogenesis.

In this regulatory context, ZNF536, located on chromosome 19q12, has emerged as a promising candidate gene. The genetic evidence implicating ZNF536 in SZ is robust. Pardiñas et al. (2018) highlighted its enrichment in SZ-associated variants (*p* < 10^−7^), predominantly in non-coding DNA (Golov et al., 2020). Subsequent analysis of the CLOZUK cohort, combined with a meta-analysis of 11,260 cases and 24,542 controls, identified multiple intronic variants within ZNF536, with rs2053079 showing the highest posterior probability (0.910). This variant resides in a region under strong back-ground selection, where low recombination rates allow slightly deleterious alleles to persist at moderate frequencies via genetic drift—a pattern observed across SZ-associated loci (Satterstrom et al., 2019). Beyond common variants, ZNF536 exhibits extreme intolerance to protein-truncating variants (PTVs), with a pLI score ≥ 0.9. Satterstrom et al. (2019) detected no PTVs in 5,002 controls or the gnomAD database (*n* = 44,779) yet identified four PTVs exclusively in individuals with neurodevelopmental disorders (*P* = 4.24 × 10^−4^), including *de novo* PTVs in ASD probands (e.g., c.1571G>A; Trp524Ter; Costa et al., 2023). This duality suggests that while common variants may subtly elevate SZ risk, rare, high-impact mutations could anchor more severe neurodevelopmental phenotypes, potentially overlapping with SZ. Apart from SZ and ASD, ZNF536 has also been implicated in a broader spectrum of psychiatric conditions through both genetic and epigenetic studies. Epigenetic regulation of ZNF536 has been associated with ADHD, particularly in the context of prenatal environmental exposures (Chaumette et al., 2023), and major depressive disorder (Lin et al., 2018), where it may influence antidepressant response. Additional associations have been reported with bipolar disorder (Winham et al., 2014; Xu et al., 2014), male sexual orientation (Hu et al., 2021), and behavioral traits related to body weight comorbidity in ADHD (Dmitrzak-Weglarz et al., 2021), further emphasizing ZNF536's pleiotropic role across neurodevelopmental and psychiatric phenotypes.

ZNF536 encodes a transcription factor with a modular structure: 10 C2H2-type zinc finger domains at the C-terminus for DNA binding and an N-terminal CtBP-binding motif (PLDLSVP) that recruits the co-repressor CtBP. This architecture enables ZNF536 to regulate gene expression through direct DNA interactions and chromatin modification. Functionally, ZNF536 negatively regulates retinoic acid (RA)-induced neuronal differentiation by binding RA response elements (RAREs), repressing neuronal marker genes like Pax6, MAP2, and β-Tubulin III in P19 cells (Qin et al., 2009). Its expression is highly conserved (approximately 90% amino acid identity between humans and mice) and brain-specific, concentrated in SZ-relevant regions such as the cerebral cortex and hippocampus, with minimal presence outside the central nervous system (Al-Naama et al., 2020). This evolutionary conservation and neuronal specificity underscore ZNF536's critical role in neurodevelopment, suggesting that its dysregulation could disrupt processes linked to SZ pathology.

The regulatory landscape of ZNF536 further amplifies its neurodevelopmental significance. Enhancer databases (e.g., VISTA) identify elements like ID:hs384.0, located in an intron between exons 2 and 3, active in the embryonic forebrain. This enhancer may also regulate TSHZ3, a neighboring gene implicated in autism and psychiatric disorders, hinting at shared cis-regulatory mechanisms that could heighten neurodevelopmental risk (Caubit et al., 2016, 2022).

Despite its consistent implication in SZ and related disorders, ZNF536's precise molecular contributions remain underexplored. GWAS signals, while suggestive, often lack genome-wide significance in larger cohorts (e.g., rs8104557 in Trubetskoy's GWAS, Supplementary materials), reflecting the challenge of detecting subtle effects in conserved genes. In contrast, rare PTVs and their enrichment in ASD and neurodevelopmental disorders highlight a spectrum of impact—from common variants with incremental SZ risk to rare mutations driving severe outcomes.

In addition, animal models provide compelling functional insights. CRISPR/Cas9-generated znf536 knockout zebrafish exhibit reduced forebrain volume, cerebellar defects (e.g., decreased Purkinje cell populations), and behavioral changes reminiscent of SZ pathology. Two complementary approaches demonstrate this: Thyme et al. (2019) screened 132 SZ -associated genes in larvae, while Kim et al. (2024) conducted longitudinal analysis of ZNF536 knockout from larval to adult stages, revealing cerebellar deficits and specific behavioral impairments. These findings confirm ZNF56's role in neuronal differentiation and development.

This work aimed at investigating how ZNF536 dysfunction contributes to SZ-pathogenesis. We employed CRISPR/Cas9 to generate ZNF536 knockout SH-SY5Y cell lines, creating two variants: a homozygous knock-in line (KI/KI) with a selection cassette disrupting exon 2, and a compound heterozygous line (KI/del) with a 103 kb deletion spanning exons 2–4, encompassing SZ-associated variants with disruption of zinc finger domains. SH-SY5Y cells, which differentiate into neurons upon ATRA treatment—a process ZNF536 regulates—offer an ideal platform to study neuronal development.

Here, we show that ZNF536 disruption impairs neuronal differentiation and alters neuro-developmental gene networks, offering mechanistic insight into its role in SZ. Transcriptome pro-filing before and after all-trans retinoic acid (ATRA) treatment revealed downregulation of RAR target genes, impaired neurite outgrowth, and dysregulation of E2F4-driven cell cycle programs. Deletion of intronic regions also affected expression of SZ-associated genes, highlighting their likely regulatory function. These findings position ZNF536 as a key modulator of RA signaling and neurogenic transcription, bridging GWAS signals with neurobiological mechanisms underlying SZ.

## Materials and methods

### Cell culturing

SH-SY5Y and HEK293T cell lines were sourced from the Biomaterial Collections Fund “NeuroResource” (https://ckp-rf.ru/catalog/usu/4145280/) at the Mental Health Research Center (Moscow, Russia). The isogenic KI/KI and KI/del mutant SH-SY5Y lines generated in this work are deposited in the same biobank. Cells were cultured in DMEM/F12 medium (Paneco LLC, Moscow, Russia) supplemented with 10% fetal bovine serum (Gibco, Waltham, MA, USA) with penicillin (50 U/mL) and streptomycin (50 μg/mL; Paneco LLC). The medium was changed every 2–3 days. Once the cell layer reached a subconfluent state, cells were separated using a 0.05% trypsin EDTA solution with Hanks' salts (Paneco Ltd., Singapore) and passaged in a 1:4 ratio. Cultures were incubated in a humidified CO2 incubator MCO-18AC (Sanyo, Osaka, Japan) at 37 °C and 5% CO2.

### Construction of plasmids

A lentiviral vector expressing SpCas9 was constructed from two plasmids, pCW-Cas9 and pEGFP-Puro, as it was designed in this work (Abashkin et al., 2023).

For targeting the ZNF536 locus, we constructed a modular sgRNA expression vector through a multi-step cloning approach. First, we amplified the U6 promoter region from pLK05 using PCR with primers U6_Bso31I_F and U6_AsiGI_R (Table 1), incorporating Bso31I and AsiGI restriction sites. This 835 bp pLK05_PCR fragment contains essential elements for sgRNA expression: the U6 RNA polymerase III promoter, gRNA scaffold sequence, a cPPT/CTS element, and a portion of the EF-1α core promoter with a Kozak sequence for efficient translation initiation. The RFP expression cassette, including the EF1α promoter, was isolated from the pCheck plasmid (Abashkin et al., 2023). This second fragment was digested with AsiGI and PciI. These two inserts were then simultaneously cloned into the PetBlue2 backbone, which had been linearized with PciI and EcoRI, creating an intermediate construct containing both the U6 promoter for sgRNA expression and the RFP marker for transfection monitoring. Subsequently, two guide RNAs targeting exon 2 (ZNF536_S1: Table 1) and exon 4 (ZNF536_S2: Table 1) were selected based on their on-target efficiency scores and minimally predicted off-target effects. The sgRNA inserts were generated by PCR amplification using blunt-end-generating polymerase with primers 204 and 205 (Table 1), which incorporate BsaI restriction sites. The resulting PCR product (ZNF536_sgRNA_insert, 356 bp) contained both sgRNA sequences, separated by a gRNA scaffold and driven by a U6 promoter. This dual-sgRNA fragment was then digested with BsaI and ligated into the BsmBI-digested intermediate vector. The final construct (sgRNA_ZNF536_in_PB2-RFP, 5128 bp) contains two expression cassettes: the dual ZNF536-targeting sgRNAs under U6 promoter control and a tagRFP reporter under EF1α promoter control, enabling visualization of successfully transfected cells. The integrity of the final construct was verified by restriction enzyme analysis and Sanger sequencing of the sgRNA regions.

**Table 1:** Oligonucleotides for molecular cloning experiments.

**Name**	**Sequence (5^′^ → 3^′^)**
U6_Bso31I_F	AGTCAATGGTCTCAAATTCGAGGGCCTATTTCCCATGATTCCTTC
U6_AsiGI_R	CATGGTGGCAGATCCCG
ZNF536_S1_F	ATCACGGCCGAGTCGGCCCA
ZNF536_S2_R	CCACTTAGAGCGACACCATC
204_F	ATATAGGTCTCTCACCGATCACGGCCGAGTCGGCCCAGTTTTAGAGCTAGAAATAGCAAG
205_R	GAGAGGGTCTCCAAACGATGGTGTCGCTCTAAGTGGCGGTGTTTCGTCCTTTCCAC
2_fusion_sp2_F	CTTCTCTGTTCATCAGGCCAGACATGGTGGCACTTTTCGGGGAAATGTG
6-to-2_R	CGGATCAAGCGTATGCAGCCG
sp2_Ple19I_F	ATAGGGCGATCGAATAAGTCCGCACTGTGACTATGCC
_sp2_R	ATGTCTGGCCTGATGAACAGAG
3_fusion_sp1_F	AGGTGTGCGGTCAGGTGTTCAGGGACCGAACCCCGCGTTTAT
5-to-3_R	CCATCATGGCTGATGCAATGC
sp1_CciNI_F	TAATATTGCGGCCGCGAGGAGCTCATCAGCCACG
sp1_R	CTGAACACCTGACCGCACACC

To facilitate efficient integration of a selection marker at the targeted genomic loci, we designed a self-cleaving knock-in vector containing an SV40-NeoR/KanR cassette flanked by the same ZNF536 target sequences (S1 and S2). This design enables simultaneous cleavage by Cas9 of both genomic DNA and the donor plasmid, promoting efficient cassette integration via NHEJ at the targeted sites.

The construction of this self-cleaving vector involved a multi-step overlap extension PCR approach. First, we amplified the NeoR/KanR cassette from pEGFP-N1 using two separate PCR reactions. For the fragment containing the Spacer 2 sequence, we used primers 2_fusion_sp2 and 6-to-2 (Table 1). In parallel, we amplified the ZNF536 targeting sequence (Spacer 2) from genomic DNA using primers sp2_Ple19I and primer_sp2_R (Table 1).

Similarly, for the fragment containing Spacer 1, we amplified the complementary section of the NeoR/KanR cassette using primers 3_fusion_sp1 and 5-to-3 (Table 1). The corresponding ZNF536 targeting sequence (Spacer 1) was amplified using primers sp1_CciNI_F and sp1_R (Table 1).

The resulting fragments were then joined using overlap extension PCR with Phusion high-fidelity DNA polymerase (Thermo Fisher Scientific). For the Spacer 2 region, we used primers 6-to-2 and Primer_sp2_Ple19I, while for the Spacer 1 region, we used primers 5-to-3 and Primer_sp1_CciNI_F. This resulted in two fragments: part_sp2 (1092 bp) and part_sp1 (897 bp), each containing a portion of the NeoR/KanR cassette flanked by a ZNF536-targeting sequence with the appropriate restriction sites.

Both fragments were digested with Bse3DI, and additionally, part_sp2 was digested with Ple19I while part_sp1 was digested with CciNI. All restriction digestions were performed using enzymes from SibEnzym (Novosibirsk, Russia), which provide high specificity and complete digestion even with complex DNA substrates. The PetBlue-RFP-sgRNAs vector was linearized with Ple19I and CciNI, followed by a dual fragment insertion ligation to create the final self-cleaving vector (S-C_vector, 5316 bp). The integrity of the assembled vector was verified by restriction enzyme analysis and Sanger sequencing of the insertion junctions.

This self-cleaving vector design enables precise integration of the NeoR/KanR cassette at ZNF536 exon 2 (S1 site) or exon 4 (S2 site) upon co-delivery with Cas9, facilitating efficient selection of successfully edited cells using G418 (400 μg/mL).

### Transfections, lentivirus assembly, and transductions

Transfections were carried out using Lipofectamine-3000 reagent (Invitrogen, Waltham, MA, USA) according to the manufacturer's protocol. HEK293T cells were grown until 50–70% confluency in ~1 day. For standard transfections, 2 μg of plasmid DNA was used per well of a 6-well plate in DMEM/F12 supplemented with 10% FBS. Transfection efficiency was evaluated by fluorescence microscopy in DPBS at 24–48 h post-transfection, based on the proportion of cells expressing fluorescent markers.

For lentivirus production, HEK293T cells were transfected with Lipofectamine-3000 in OptiMEM-I supplemented with 0.4 μg/mL Polybrene (Sigma-Aldrich, St. Louis, MO, USA). The target plasmid and pLP packaging plasmids were combined in the following proportions: 5.8 μg target vector, 1.35 μg pLP1, 0.9 μg pLP2, and 1.33 μg pLP/VSVG. At 18–24 h post-transfection, the medium was replaced with DMEM/F12 containing 10% FBS, and virus-containing supernatant was collected at 48 and 72 h post-transfection. The supernatant was diluted 1:2 with fresh medium, filtered through a 0.45-μm PES filter, and used immediately for transduction procedures. SH-SY5Y cells were transduced according to the spinfection protocol with the addition of 10 μg/mL polybrene. For stable Cas9-expressing cell lines, transduced cells were selected with puromycin (2 mg/L) for 2–3 weeks until resistant colonies emerged.

For PetBlue2-RFP-ZNF536 sgRNA and self-cleaving vector transfections, we employed the calcium phosphate precipitation method to achieve higher transfection efficiency. Cells were plated in 6-well plates 4–6 h prior to transfection. For each well, 3–5 μg of plasmid DNA was combined with 16 μL of 2M CaCl_2_ and nuclease-free water to a final volume of 125 μL. This mixture was added dropwise to 125 μL of 2 × HEPES-buffered saline (HBS: 280 mM NaCl, 10 mM KCl, 1.5 mM Na_2_HPO4, 12 mM dextrose, 50 mM HEPES, pH 7.05) while vortexing gently. After 15–20 min of incubation at room temperature to allow precipitate formation, the transfection mixture was distributed evenly over the cells. Culture medium was replaced with fresh, pre-warmed medium 16–20 h post-transfection. Cells that had successfully integrated the NeoR/KanR cassette were selected using G418 (400 μg/mL, Sigma-Aldrich) for 2–3 weeks.

### Differentiation of SH-SY5Y by RA treatment

We employed a two-step differentiation protocol modified from Shipley et al. (2016). SH-SY5Y cells were seeded on 10-cm plates and incubated to achieve 30% confluency in DMEM/F12 medium containing 10% FBS. For neuron induction, the medium was replaced with DMEM containing 1% FBS and 10 μM ATRA. Cells were maintained in this medium for 7 days with changes every 48 h. For differentiation, cells were seeded into 10-cm Matrigel-coated plates and cultured in Neurobasal supplemented with B27, 10 μM ATRA, 50 ng/mL BDNF, and 20 mM KCl for an additional 7 days. This regimen of differentiation caused cell cycle arrest and neuronal morphological differentiation, with extensive neurite outgrowth and increased synapse formation.

### Fluorescence microscopy

Fluorescent and phase contrast images were acquired using a Nikon Eclipse Ts2 microscope equipped with a Nikon DS-Fi3 camera (Nikon, Tokyo, Japan). Cells were visualized in DPBS buffer. GFP fluorescence was detected using fluorescence filter set #3 “GFP” (excitation 446–486 nm/emission 500–550 nm), while tagRFP fluorescence was captured using fluorescence filter set #5 “mCherry” (excitation 542–582 nm/emission 604–678 nm).

### RT-qPCR analysis

Total RNA was reverse transcribed using the 5X RT MasMIX-30100 kit (DIALAT Ltd., Moscow, Russia) according to the manufacturer's protocol. For each reaction, 5 μL of 5X RT MasMIX was combined with oligo dT primers (50 μM, 0.2–0.5 μL) and RNA template (1 pg−1 μg of total RNA) in a final volume of 25 μL. Reverse transcription was performed using the following thermal cycling conditions: 25 °C for 10 min, 42–50 °C for 30 min, followed by enzyme inactivation at 85 °C for 5 min.

Quantitative PCR was performed using the 5X MasCFETaqMIX-2025 with EvaGreen dye (DIALAT Ltd., Moscow, Russia) on a QuantStudio 7 Flex Real-Time PCR System (Thermo Fisher Scientific). Each 25 μL reaction contained 5 μL of 5X MasCFETaqMIX, 0.2–1.0 μM of each gene-specific primer, and 2 μL of cDNA template (≈10 ng RNA-equivalent per reaction). Gene-specific primers were used to amplify and quantify the following targets: FOXM1, and ASCL1 (Table 2). PCR amplification was conducted using the following cycling parameters: initial denaturation at 94 °C for 3 min, followed by 40 cycles of denaturation at 94 °C for 30 s, annealing at 60 °C for 30 s, and extension at 72 °C for 30 s, with a final extension at 72 °C for 5 min. A melting curve analysis was performed from 60 °C to 95 °C to verify amplification specificity.

**Table 2:** RT-qPCR oligonucleotides.

**Name**	**Sequence (5' → 3')**	**Amplicon length (bp)**
ZNF536_spacer1_F	GGA GGA GCT CAT CAG CCA CG	155
ZNF536_spacer1_R	TGT GAC CCT TGA GGA ACC ACG	
ZNF536_exon_junc_F	CAT GAA GGA CTG CCC GTA CTG T	114
ZNF536_exon_junc_R	GCA TAG TCA CAG TGC GGA CAC T	
FOXM1_F	CGTCGGCCACTGATTCTCAAA	96
FOXM1_R	GGCAGGGGATCTCTTAGGTTC	
TSHZ3_F	GTGACCGAATGGCTGACTTTG	100
TSHZ3_R	AGGCTATCCGACACAGTCGT	
RARβ_F	TCCGAAAAGCTCACCAGGAAA	125
RARβ_R	GGCCAGTTCACTGAATTTGTCC	
ASCL1_F	AACTACTCCAACGACTTGAACTCCATGGC	84
ASCL1_R	GCTGAGCGGGTCGTAAGAGCC	
GAPDH_F	GACAGTCAGCCGCATCTTCT	93
GAPDH_R	GACCAAATCCGTTGACTCCGA	

Relative gene expression was calculated using the comparative CT (ΔΔCt) method, with GAPDH serving as the reference gene for normalization. All samples were run in triplicate, and the specificity of amplification was confirmed by melting curve analysis. Data were processed using QuantStudio Design & Analysis Software v1.5.1 (Thermo Fisher Scientific). Relative expression levels were normalized to control samples (NTC) and presented as mean ± standard deviation from three independent biological replicates. Statistical significance was determined using one-way ANOVA with Dunnett's *post-hoc* test, with *p* < 0.05 considered statistically significant.

### RNA isolation and RNA-seq library preparation

Total RNA was extracted using phenol-guanidine-isothiocyanate ExtractRNA reagent (Evrogen, Moscow, Russia) according to the manufacturer's protocol. Isolated RNA was solubilized in nuclease-free water containing RNase inhibitor (Syntol, Moscow, Russia). Residual genomic DNA contamination was eliminated using DNase I treatment (NEB, Ipswich, MA, USA). RNA integrity was assessed using the Qubit RNA IQ Assay Kit on a Qubit 4 fluorimeter (Thermo Fisher Scientific). Only RNA samples with integrity scores exceeding 7.0 were utilized for subsequent analyses.

RNA-seq library construction was conducted in a two-step process: first, polyadenylated RNA was enriched using the NEBNext Poly(A) mRNA Magnetic Isolation Module (NEB); second, the isolated polyA RNA was processed using the NEBNext Ultra II Directional RNA Library Prep Kit for Illumina (NEB). Quality control and sequencing were performed by Novogene (China). Quality assessment revealed an average library size of 283 bp, with sequencing yielding approximately 12 million reads per sample (3 GB of data per sample).

### RNA-seq data analysis

Raw reads underwent trimming using FastP (Chen et al., 2018) and quality assessment with FastQC (Wingett and Andrews, 2018). The processed reads were aligned to the Ensembl reference genome (GRCh38/hg38; Nurk et al., 2022) in pairwise mode using the STAR aligner (v2.7.10b; Dobin et al., 2013) on the Yandex cloud platform. Resulting alignments were subjected to deduplication via the Picard tool suite 9 (v2.27.4; Picard Tools, 2023). Gene-level counting was performed using HTSeq (v2.0.2; Anders et al., 2015), and differential expression analysis was conducted with DESeq2 (v1.38.3; Love et al., 2014) in R (BioConductor package). GSEA was performed on DESeq2 results using the fgsea package (v1.24.0; Korotkevich et al., 2019; adjusted *p*-value < 0.05), querying the MSigDB and Hallmark databases (v2023.1; Liberzon et al., 2015). DisGeNET (v7.0; Piñero et al., 2021), Gene Ontology, and functional enrichment analyses were conducted using the DAVID online service (v2023; Huang et al., 2009). All enrichment analyses were performed against the background of the entire transcriptome generated within the same experimental context. Transcription factor enrichment analysis utilized the ChEA3 online service (Keenan et al., 2019). ChIP-seq data for RARE were obtained from http://chip-atlas.org/ and analyzed for genomic overlaps within ±2 kb of transcription start sites using the “bedtools intersect” command.

### Cell migration assays

Cell migration capacity was evaluated using wound healing assays in starvation medium (growth medium containing 0.1% FBS). Cells were cultured to near-confluence in 35-mm dishes before creating five parallel scratches per dish using a micropipette tip. Ten images per plate were captured immediately after scratching (0 h) and at subsequent time points (6 and 24 h). Wound closure was quantified by measuring gap width using ImageJ with the Wound_healing_size_tool plugin (Suarez-Arnedo et al., 2020).

### Neurite outgrowth assays

Cells were seeded at densities empirically optimized so that untreated and ATRA-treated cultures reached comparable confluency at the end of the 7-day differentiation period. All-trans retinoic acid (ATRA, 10 μm) was applied for 7 consecutive days; culture medium was refreshed every 48 h with freshly prepared ATRA. For each sample, ≥5 non-overlapping fields were captured. Images were calibrated to the microscope scale, and neurite lengths were quantified in ImageJ/Fiji by NeuronJ plugin (Popko et al., 2009).

## Results

### CRISPR-Cas9-mediated generation and characterization of ZNF536 mutant lines

**Figure 1:**
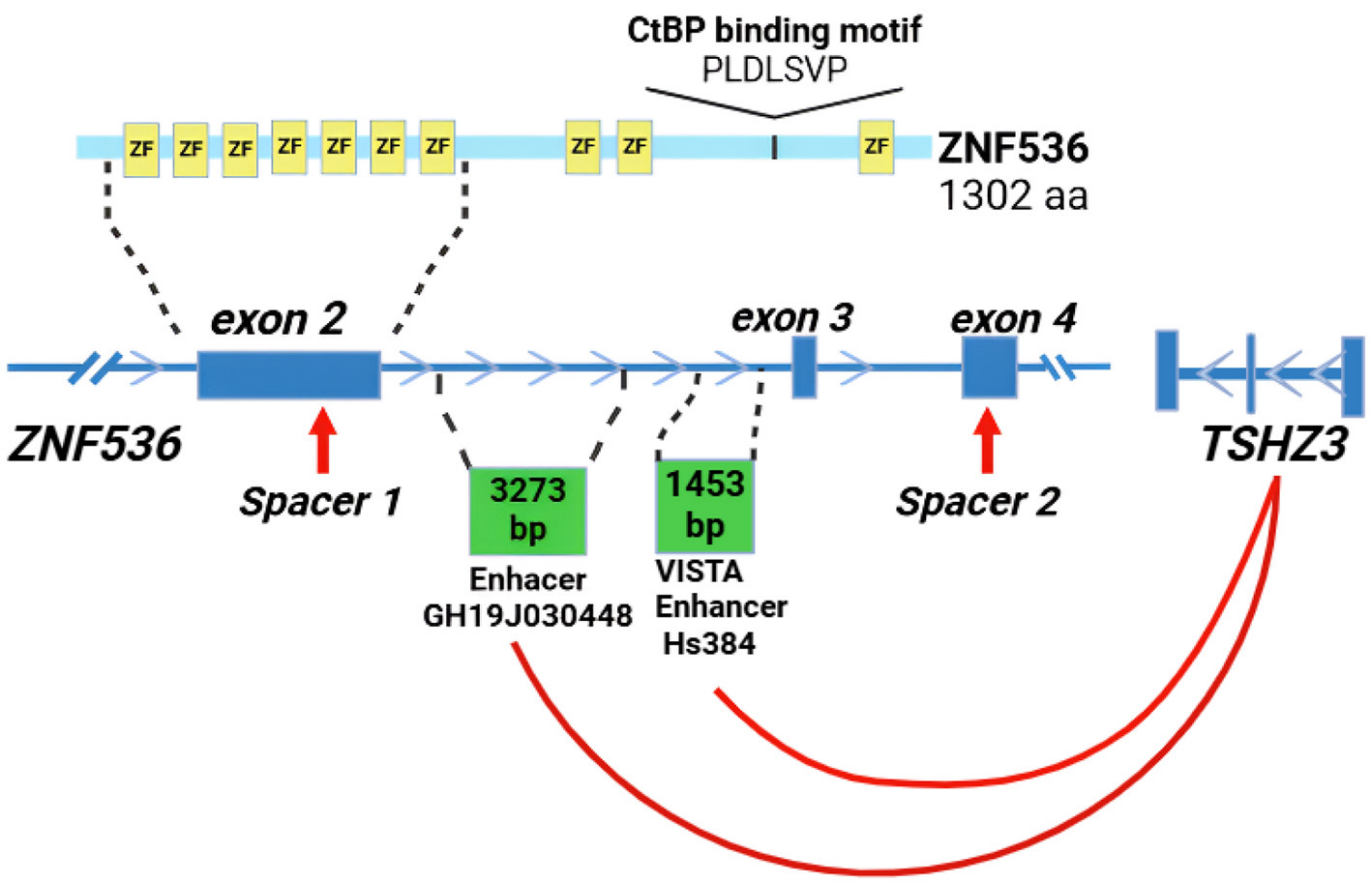
Structure of ZNF536 protein and gene locus. Schematic illustrating the ZNF536 protein (1,302 amino acids), with its 10 C2H2-type zinc finger domains (ZF, yellow) and a CtBP-binding motif (PLDLSVP), aligned with the genomic structure spanning exons 2–4 (blue). Two potential intronic enhancers—GH19J030448 (3,273 bp) and VISTA Enhancer hs384 (1,453 bp)—are positioned downstream of exon 2 (green boxes). These regulatory elements may influence expression of ZNF536 as well as the adjacent gene TSHZ3, which has been implicated in neurodevelopmental disorders. CRISPR-Cas9 target sites (red arrows, labeled Spacer 1 and Spacer 2).

GWAS revealed significant enrichment of SZ-associated variants within the ZNF536 locus (*p* < 10–7), notably in a genomic region spanning exons 2–4 that contains candidate cis-regulatory elements (GH19J030448, hs384.0; Kosicki et al., 2025). As illustrated in Figure 1, our initial aim was to delete the ~103 kb interval encompassing these exons. PCR screening utilized primers flanking that large region, ensuring that only successful deletion events would yield amplification products. Initial CRISPR-Cas9 editing efforts produced heterozygous deletions (del/+), confirmed by Sanger sequencing and quantitative PCR that showed a reduced ZNF536 copy number.

**Figure 2:**
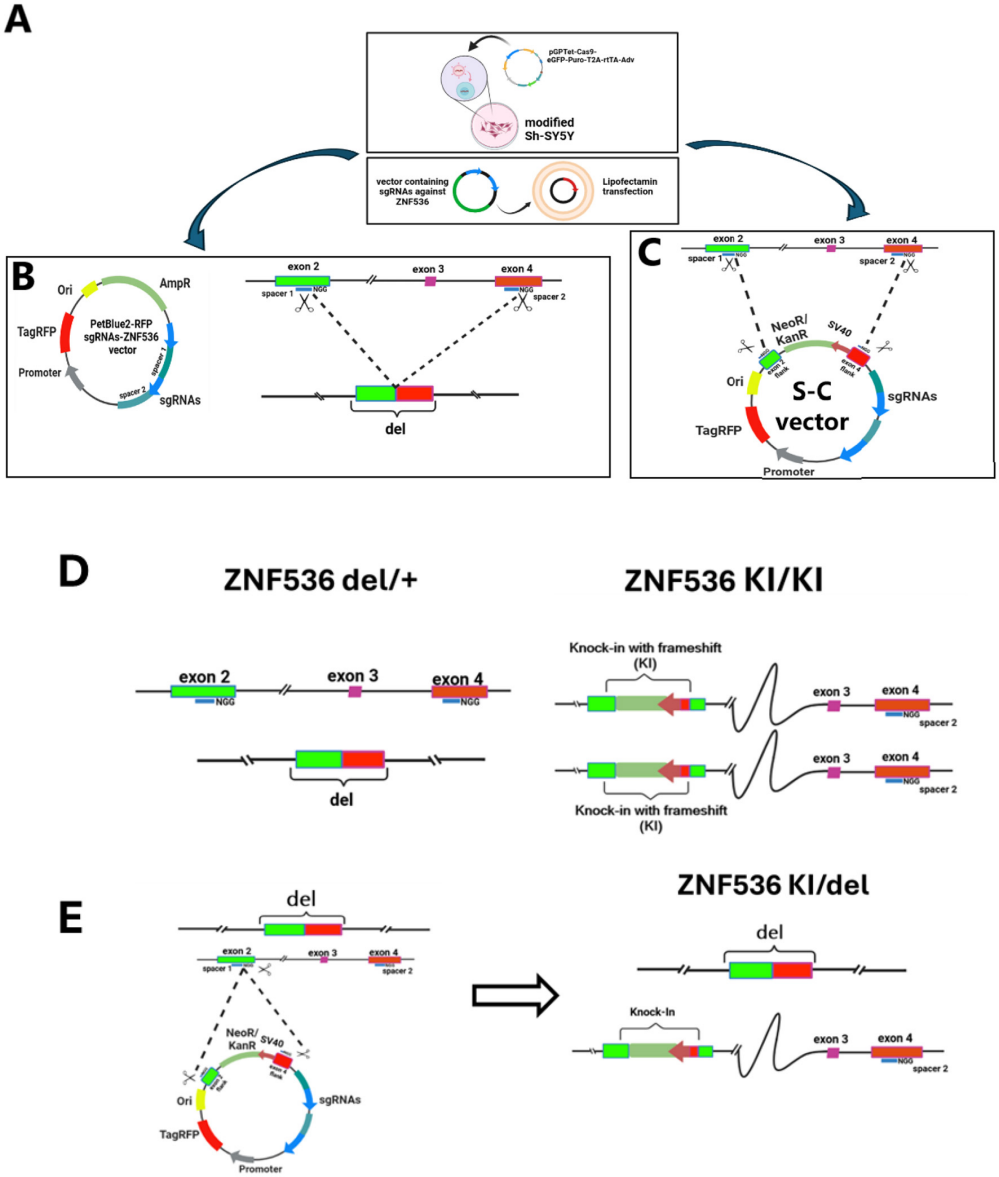
Design of CRISPR-Cas9 editing vectors **(A)**. PetBlue2-RFP vector containing sgRNAs targeting ZNF536 exons 2 and 4, enabling large deletion (del) **(B)**. Self-cleaving vector design incorporating an SV40-NeoR/KanR cassette flanked by the same sgRNA target sequences for knock-in (KI) generation. Modified SH-SY5Y cells expressing pGPTet-Cas9-eGFP-Puro-T2A-rtTA-Adv were transfected with these vectors using Lipofectamine **(C)**. Generation of heterozygous deletion (del/+) and homozygous knock-in (KI/KI) lines. Left: del/+ configuration showing one wild-type allele and one allele with ~103 kb deletion between exons 2–4. Right: KI/KI configuration showing homozygous integration of the NeoR/KanR cassette at exon 2, with intact downstream exons **(D)**. Strategy for generating compound heterozygous (KI/del) mutants. The self-cleaving vector was introduced into del/+ cells, resulting in cassette integration at exon 2 of the remaining wild-type allele, creating cells with both deletion and knock-in modifications on separate alleles **(E)**. NGG, PAM sequence; del, deletion; KI, knock-in. Colored boxes represent exons and regulatory elements. Dashed lines indicate CRISPR-mediated modifications.

During population screening, we observed rare integration events in which an ~800 bp random fragment of plasmid backbone was incorporated between guide RNA target sites via non-homologous end joining (NHEJ). This led us to develop a self-cleaving vector strategy, where an SV40–NeoR/KanR (G418 resistance) cassette was flanked by the same ZNF536 spacer sequences. By co-delivering Cas9 and this self-cleaving vector, we enabled simultaneous cleavage of both genomic and plasmid-borne sites (Figure 1), facilitating cassette integration via NHEJ. When introduced into modified SH-SY5Y cells expressing pGPTet-Cas9-eGFP-Puro-T2A-rtTA-Adv (Abashkin et al., 2023), the G418 cassette consistently integrated at the exon 2 (S1) site, whereas editing at exon 4 (S2) was rarely detected. This result yielded homozygous knock-in (KI/KI) clones specifically at the exon 2 locus (Figure 2). Several factors may account for this outcome, including differences in local chromatin accessibility or variable repair pathway efficiencies favoring cassette integration at exon 2 over exon 4. Moreover, partial disruption at one allele of a gene with high loss-of-function (LoF) intolerance (pLI = 1.0) could exert selective pressure, reducing the likelihood that a second damaging event would be tolerated in the same cells.

To achieve complete inactivation of ZNF536, we next applied the self-cleaving vector system to the del/+ cells, creating compound heterozygous (KI/del) mutants in which one allele carried the 103 kb deletion, while the remaining wild-type allele was targeted for G418 cassette insertion. In previous screening of clones, we isolated one mutant with heterozygous deletion of ZNF536 out of 80. This strategy leveraged allele-specific differences in editing outcomes: although cells did not tolerate simultaneous disruption of both ZNF536 copies at once, they survived a stepwise approach—first generating del/+ cells, then targeting the second allele. Altogether, these new SH-SY5Y lines—del/+, KI/KI, and KI/del—constitute a robust allelic series for dissecting ZNF536 function and its associated SZ-linked regulatory elements. By demonstrating how sequential or allele-specific manipulations can circumvent cellular lethality in the face of strong LoF intolerance, our experiments establish an effective strategy for achieving complete gene inactivation while preserving cell viability. Rather than posing a limitation, allele-specific editing thus emerges as a practical approach to generate null genotypes in genes otherwise essential for cell survival.

### Phenotypic differences during RA-induced differentiation

**Figure 3:**
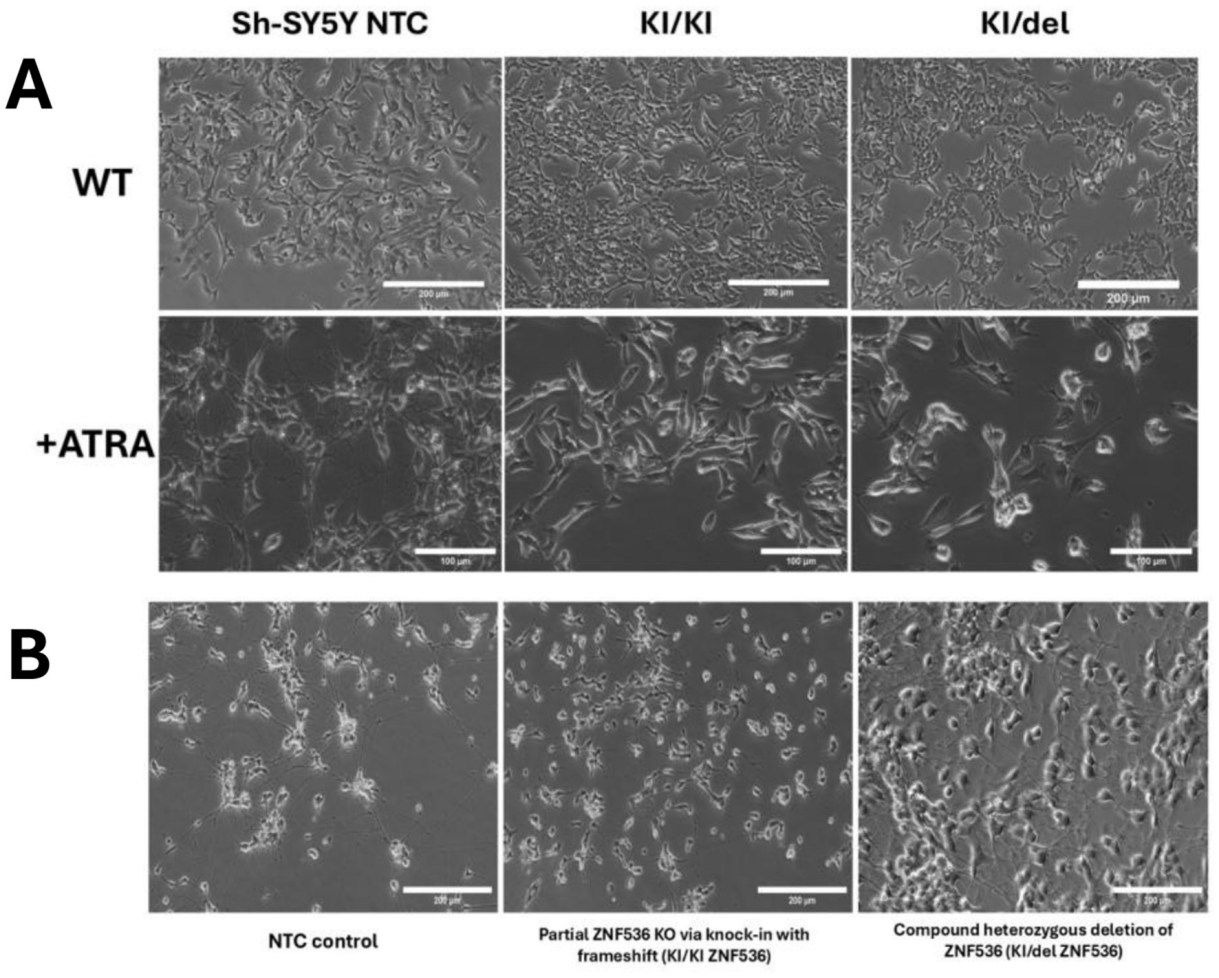
ZNF536 is required for ATRA-induced neuronal differentiation in SH-SY5Y cells. **(A)** Phase-contrast images of control (NTC) and ZNF536 mutant lines (KI/KI, KI/del) under basal conditions **(top)** and after 10 days of ATRA treatment **(bottom)**. **(B)** Neuronal maturation following BDNF/ATRA- treatment on Matrigel-coated plates. NTC cells maintain complex neuritic networks, while mutants show rescued neuronal phenotypes. Scale bar: 200 and 100 μm.

SH-SY5Y cells exhibit a well-characterized ability to differentiate into neuron-like cells upon retinoic acid (RA) treatment (Kovalevich and Langford, 2013) a process regulated in part by ZNF536. Consequently, SH-SY5Y provides a robust *in vitro* platform to directly observe the functional impact of ZNF536 loss on neuronal differentiation and maturation, complementing the systemic insights gained from animal studies. We employed a two-step differentiation protocol (Shipley et al., 2016; see section Materials and methods) in which SH-SY5Y cells were first treated with ATRA in low-serum medium (1% FBS) for 7–10 days (Differentiation Media #1 and #2). In the second stage, cells were transferred to ECM-coated dishes and supplemented with BDNF and db-cAMP (Differentiation Media #3) to enhance neuronal maturation. After 10 days of ATRA exposure, control SH-SY5Y cells (NTC) exhibited extensive neuritic outgrowth, characterized by long, branched processes and abundant cell–cell connections (Figure 3, left). In contrast, ZNF536 mutant lines showed markedly impaired differentiation: KI/KI mutants remained predominantly rounded with minimal neurite formation (few short processes) and KI/del mutants displayed an intermediate phenotype, with partial neurite extension but significantly reduced complexity compared to NTC (Figure 3A, middle and right). These morphological differences were consistent across three independent experiments (*n* = 3), indicating that ZNF536 is essential for RA-driven neuronal differentiation in SH-SY5Y cells.

**Figure 4:**
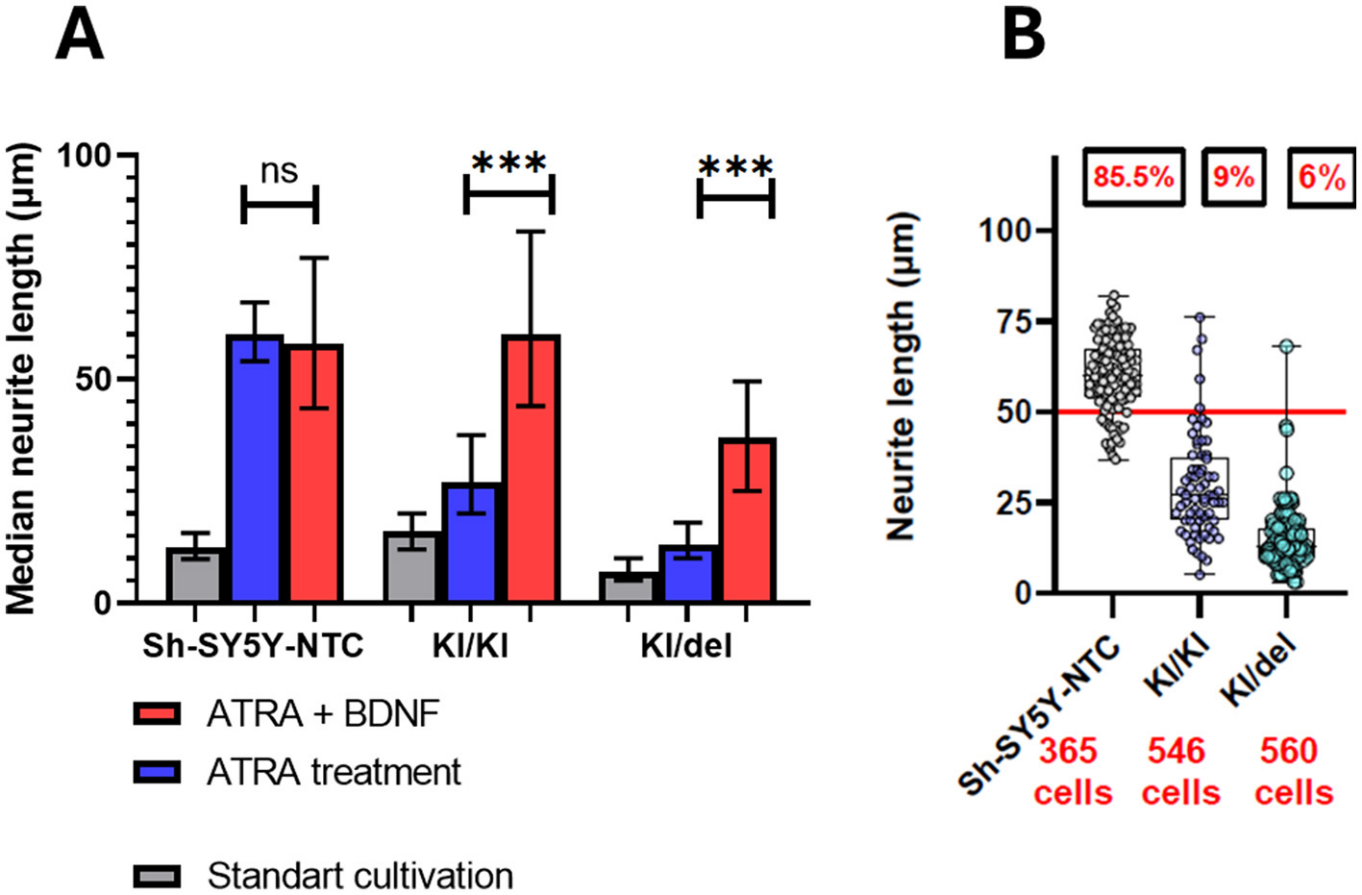
**(A)** Neurite lengths were measured using the NeuronJ plugin for ImageJ, and the median length was calculated for each replicate (n=3 independent experiments). Medians are plotted with error bars representing IQR. Statistical significance was determined using one-way ANOVA with Tukey's *post-hoc* test (***p < 0.001; ns, not significant). **(B)** Lengths of neurite outgrowths in ATRA-treated cells (data from all replicates combined) are plotted as a scatter plot. The horizontal red line marks the 50 μm threshold, an established marker of differentiation. The percentage of neurites longer than 50 μm and the total number of cells analyzed are indicated above each group in red.

To further promote maturation, cells were plated on ECM-coated (Matrigel) surfaces and treated with BDNF and ATRA for 4–7 days. While NTC cells maintained complex neuritic networks with highly branched processes (Figure 4, left), mutant lines showed partial rescue: KI/KI and KI/del mutants exhibited improved neurite outgrowth compared to ATRA-only treatment. This partial recovery suggests that ECM support and neurotrophic factors (BDNF) can partially compensate for ZNF536 deficiency but are insufficient to restore wild-type neuronal morphology.

Then we measured neurite lengths using ImageJ software and quantified our observed differences due to the NeuronJ plugin for ImageJ (Popko et al., 2009). In standard cultivation conditions without ATRA, median neurite lengths were minimal and comparable across all cell lines (Figure 3). ATRA treatment alone significantly increased median neurite lengths in NTC cells but resulted in only modest, non-significant increases in KI/KI and KI/del mutants. The addition of BDNF to ATRA further enhanced outgrowth in NTC cells and provided partial rescue in mutants, though lengths remained lower than in NTC.

Our quantitative analysis demonstrates that ZNF536 mutant cell lines exhibit significantly impaired responses to ATRA treatment alone compared to control cells. Under standard differentiation conditions, only 9% and 6% of KI/KI and KI/del cells, respectively, achieved neurite lengths above the 50 μm differentiation threshold, compared to 85.5% of control. Statistical analysis revealed that mutant lines require additional neurotrophic support beyond ATRA alone to achieve meaningful differentiation responses. While ZNF536 KO via KI severely impairs neurite formation in standard RA-only conditions, the combination of ECM support along with BDNF can partially rescue the neuronal phenotype. This suggests that alternative signaling pathways may compensate for ZNF536 deficiency during later stages of differentiation, though they cannot fully restore wild-type morphology and connectivity. It indicates that ZNF536 likely regulates early steps of neuronal differentiation via RARs co-regulation, while downstream processes can be partially activated through ZNF536-independent mechanisms when provided with appropriate environmental cues.

### Migration assay confirms functional impairments

**Figure 5:**
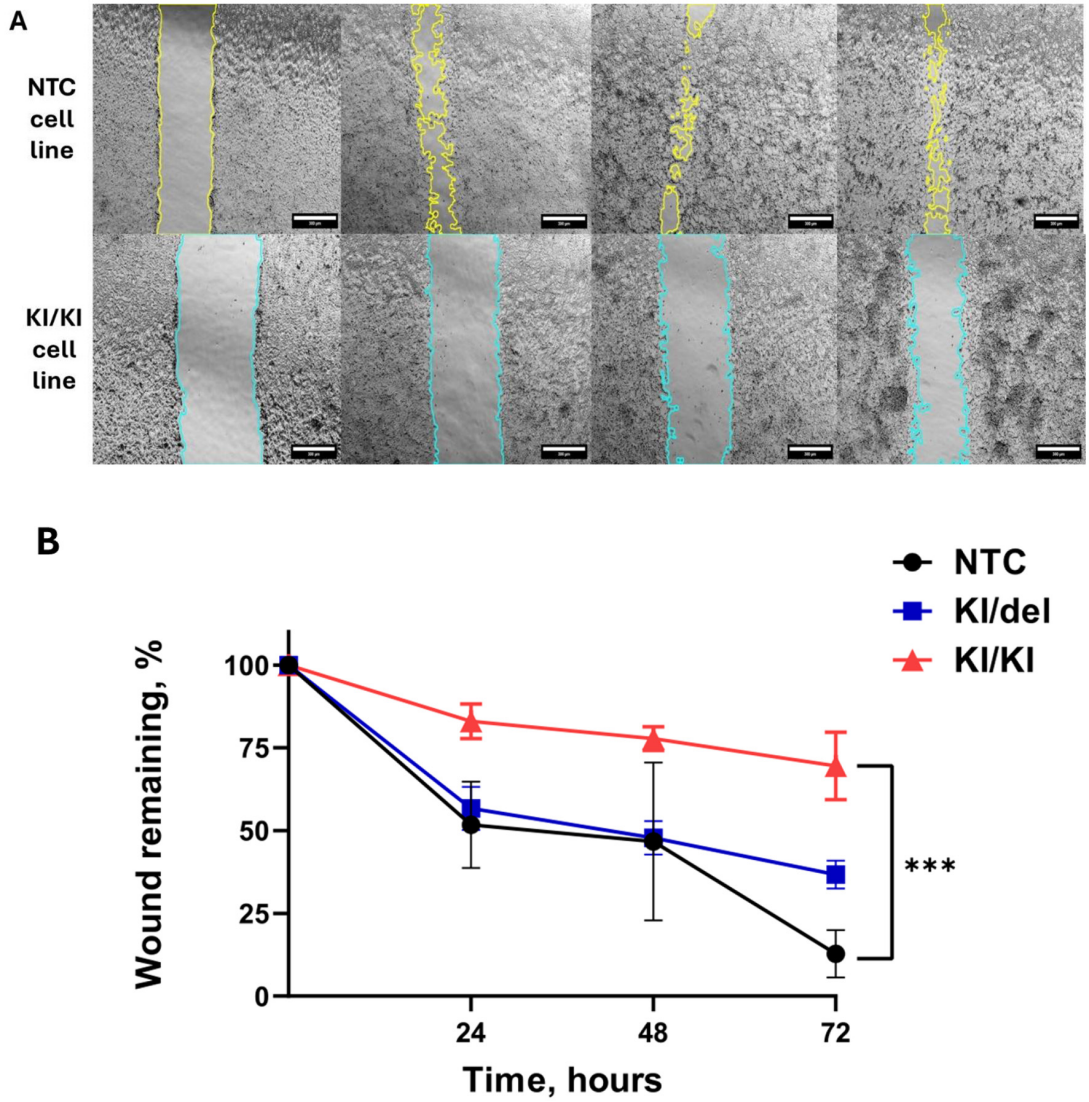
ZNF536 regulates cell migration in SH-SY5Y cells. Representative phase-contrast images from a scratch-wound assay in control (NTC) and KI/KI mutant lines at 0, 24, 48, and 72 h post-scratch **(A)**. Cell-free wound edges are highlighted in color for clarity (yellow for NTC, blue for KI/KI). Scale bar: 300 μm. Quantification of wound closure over time **(B)**. The *y*-axis represents the percentage of initial wound area remaining at each time point (mean ± SD; *n* = 10 biological replicates per condition). At 72 h, pairwise comparisons between all groups were significant (***p < 0.001, Tukey's *post-hoc* test).

To assess whether these morphological differences such as impaired neurite outgrowth corresponded to functional changes, we performed a wound healing assay on confluent monolayers of control and mutant cells (Figure 5). By 72 h post-scratch NTC cells showed robust migration, closing ~85% of the wound while KI/KI mutants exhibited significantly impaired wound closure (~30%). In turn, KI/del mutants demonstrated intermediate closure (~60%). These trends were reproducible across three experiments (mean ± SD, *p* < 0.001), underscoring the importance of ZNF536 in modulating both neuronal differentiation and cell migration.

### ZNF536 modulates expression of key neuronal differentiation genes

To validate the functional impact of our CRISPR modifications, we first confirmed G418 resistance marker integration at the ZNF536 locus through PCR amplification and Sanger sequencing (primer sequences listed in Supplementary Table S1). Both KI/KI and KI/del lines showed stable integration with premature stop codons immediately following the coding sequence of exon 2, which contains ZNF56's critical C2H2-sites.

**Figure 6:**
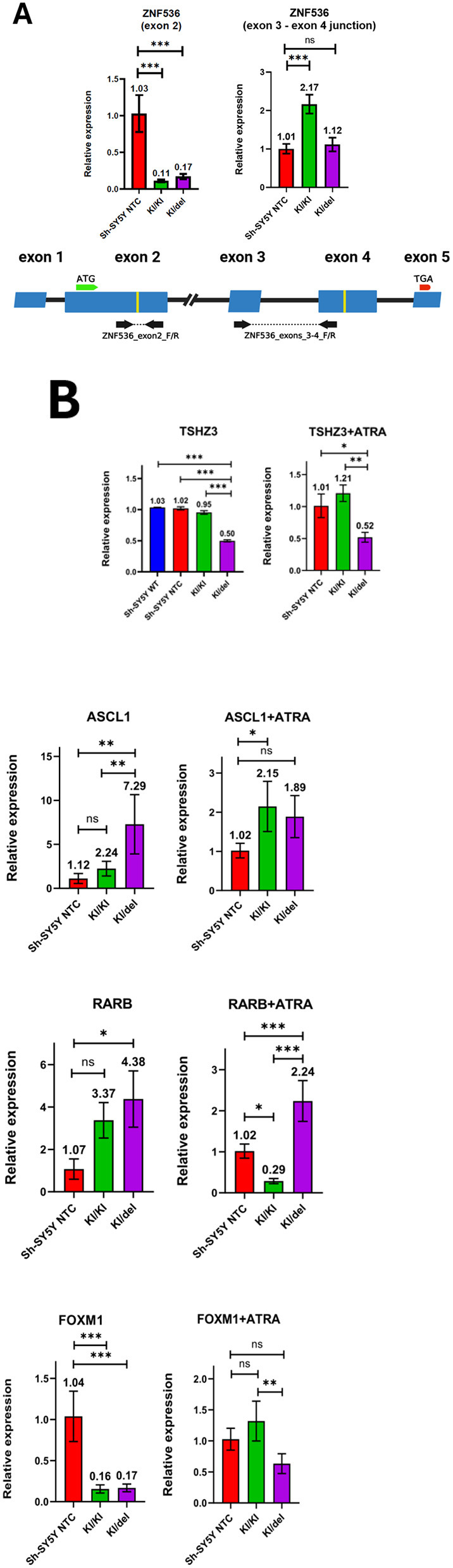
*Expression analysis of neuronal regulatory genes in ZNF536 mutant lines*. RT-qPCR analysis. **(A)** RT-qPCR analysis of ZNF536 expression using two primer sets. Left panel shows expression of the canonical ZNF536 transcript using primers targeting exon 2, revealing significant downregulation in both KI/KI (0.11-fold) and KI/del (0.17-fold) mutant lines compared to control (Sh-SY5Y NTC). Right panel shows expression of an alternative splice variant using primers spanning the exon 3–4 junction, demonstrating compensatory upregulation in KI/KI cells (2.17-fold) with lower expression in KI/del cells (1.12-fold). The gene structure diagram below illustrates ZNF536 exon organization with primer locations indicated by arrows. Yellow bars mark sgRNA target sites. **(B)** Comparison of gene expressions of four key neuronal regulatory genes (TSHZ3, ASCL1, RARB, and FOXM1) in NTC, KI/KI, and KI/del SH-SY5Y cells under standard conditions and after 7-day ATRA treatment (1% FBS). Expression values were normalized to GAPDH and shown relative to NTC (set as 1.0). Data represents mean ± SD from three independent experiments. Statistical significance was determined using one-way ANOVA with Dunnett's *post-hoc* test (*p < 0.05, **p < 0.01, ***p < 0.001, ns, not significant).

RT-qPCR analysis using primers spanning the sgRNA target region (exon 2; Supplementary Table S1) revealed dramatic downregulation of ZNF536 expression in both mutant lines: KI/KI showed 89% reduction and KI/del showed 83% reduction compared to control cells (Figure 6A). To address potential splice variant compensation, we designed primers spanning the exon 3–4 junction (Supplementary Table S1) and detected upregulation in both mutant lines, with KI/KI showing 2.17-fold increase (Figure 6A). This compensatory upregulation may presume activation of a cryptic promoter upstream of exon 3 in response to canonical transcript disruption. The expression pattern is consistent with the predicted X43 isoform (XM_017027543.3) that initiates translation from exon 3. Sanger sequencing confirmed the expected transcript structure (Supplementary Figure S1). To note, the lower alternative isoform expression in KI/del (1.12-fold) compared to KI/KI (2.17-fold) directly reflects the heterozygous deletion of the exon 2–4 region in the KI/del line. While this truncated isoform may produce protein, it lacks the essential N-terminal zinc finger domains encoded by exon 2 that are required for DNA-binding function. Therefore, both mutant lines represent functional ZNF536 loss-of-function models despite potential residual protein production.

Then we selected four key genes for expression analysis based on their established roles in neurodevelopment and potential regulatory relationships with ZNF536. This we examined how different genotypes (KI/KI vs. KI/del) affect neuronal differentiation process:

1. RARB—A direct mediator of RA signaling, chosen to assess ZNF56's role in RA-dependent differentiation (Berenguer and Duester, 2022)
2. ASCL1—A master regulator of neurogenesis, selected to evaluate effects on neural progenitor commitment (Parkinson et al., 2022)
3. TSHZ3—A neurodevelopmental transcription factor located near ZNF536, included to examine potential enhancer-mediated regulation (Caubit et al., 2016, 2022)
4. FOXM1—A proliferation-associated factor, chosen to monitor cell cycle-related changes during differentiation (Sullivan et al., 2012; Grant et al., 2013)

The results of RT-PCR are presented on the Figure 6.

These expression patterns reveal three key findings: First, the intronic region containing GH19J030448 and hs384.0 appears necessary for normal TSHZ3 expression, as evidenced by the specific reduction in KI/del cells (Figure 6). Second, proper RARB induction requires intact coding sequence, demonstrated by the impaired response in KI/KI cells (Figure 6). Third, the intronic region contains elements affecting multiple genes, as shown by the combined effects on TSHZ3 and ASCL1 expression in KI/del cells (Figure 6). Additionally, basal FOXM1 expression is markedly reduced in mutant lines, a finding consistent with their observed migration deficits, suggesting FOXM1 downregulation may contribute to altered cellular motility (Figure 6).

### General characterization of transcriptomes of SH-SY5Y cell lines

These pronounced transcriptional divergences between mutant and control lines—particularly under RA-induced differentiation—suggest that ZNF536 disruption alters pathways critical to neuronal maturation. To delineate the molecular mechanisms underlying these global expression shifts, we leveraged RNA-seq data from NTC, KI/KI, and KI/del lines under both untreated and ATRA-treated conditions to perform differential gene expression (DEG) analysis.

**Figure 7:**
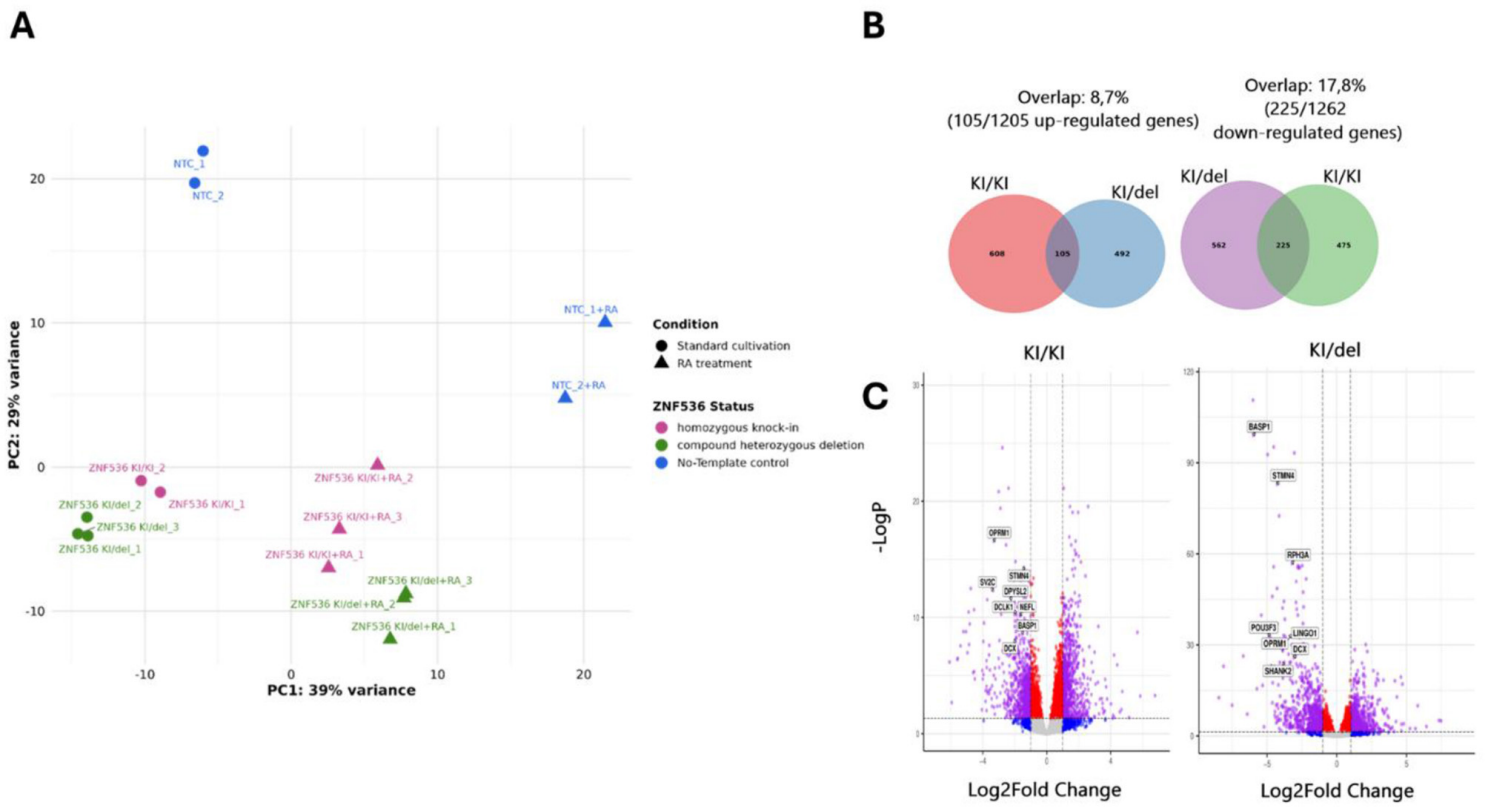
Transcriptome analysis of ZNF536 mutant SH-SY5Y cell lines under standard growth conditions. Principal component analysis (PCA) plot showing sample clustering based on RNA-seq data. The first principal component (PC1, 39% variance) separates RA-treated from untreated samples, while the second component (PC2, 29% variance) distinguishes NTC from ZNF536 mutant lines. Triangles represent biological replicates; colors indicate different genotypes and treatment conditions **(A)**. Venn diagrams showing overlap of differentially expressed genes (DEGs) between KI/KI and KI/del lines cultured under standard conditions. Left: upregulated genes (105 shared, 8.7% overlap); Right: downregulated genes (225 shared, 17.8% overlap). Numbers in parentheses indicate total DEGs specific to each condition **(B)**. Volcano plots display differential gene expression in KI/KI **(left)** and KI/del **(right)** compared to NTC under standard conditions. Red dots represent significantly altered genes (adjusted *p*-value < 0.05, |log2FC| > 1); blue dots indicate genes below significance or fold-change thresholds. Labeled genes represent key neuronal markers that show significant expression changes in both mutant lines, including NEFL (axonal structure), BASP1 (neurite outgrowth), and STMN4 (microtubule dynamics) **(C)**. The –log10(adjusted *p*-value) is plotted against log2 fold change. Gene expression changes were considered significant at adjusted *p*-value < 0.05 using DESeq2 analysis with Benjamini-Hochberg correction.

Principal component analysis demonstrated distinct clustering patterns, with the first principal component (39% variance) separating RA-treated from untreated samples, and the second component (29% variance) distinguishing NTC from mutant lines (Figure 7).

**Figure 8:**
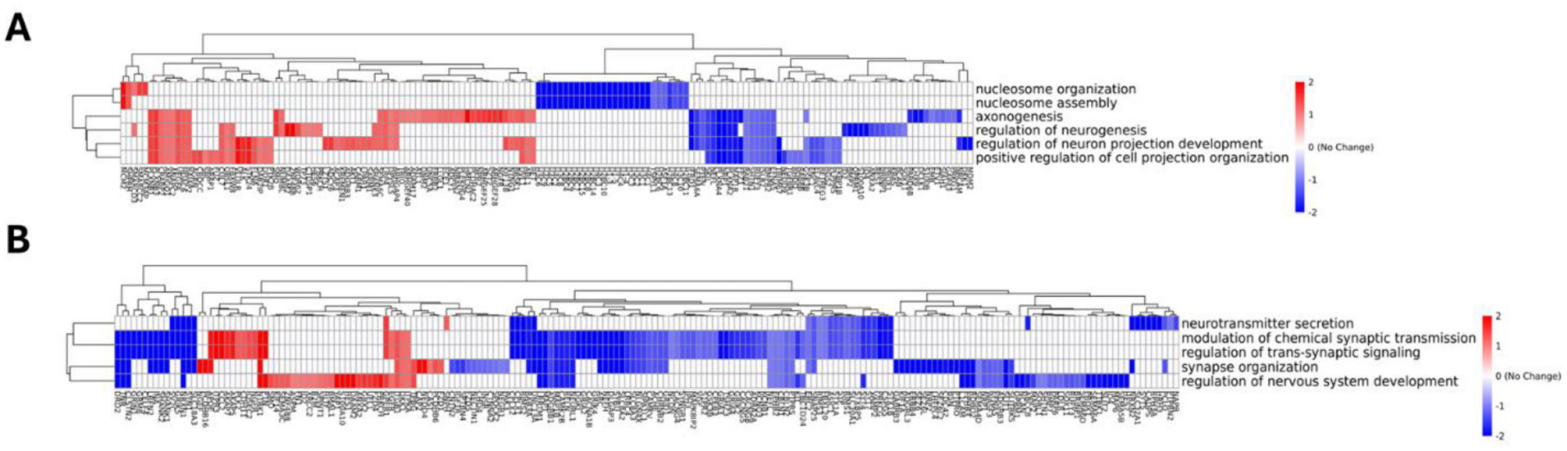
GO Enrichment Analysis Reveals Distinct Regulatory Programs in ZNF536 Mutant Lines. Hierarchical clustering heatmaps show differential regulation of GO terms in ZNF536 KI/KI **(A)** cells showing disruption of chromatin organization and neuronal development pathways, and ZNF536 KI/del **(B)** cells displaying coordinated downregulation of synaptic function and nervous system development pathways. Red indicates upregulation, blue indicates downregulation (log2 fold change scale from −2 to +2). Cells were analyzed under standard growth conditions. Rows represent GO terms, columns represent differentially expressed genes, and dendrograms show hierarchical clustering relationships.

Among significantly altered genes, we identified three distinct functional categories (Figure 8):

- Cytoskeletal components (NEFL, BASP1, STMN4) showed consistent changes across both variants
- Neurodevelopmental regulators displayed variant-specific alterations (DCX, DPYSL2 in KI/KI; SHANK2 in KI/del)
- Signaling proteins exhibited mutation-specific changes (OPRM1 in KI/KI; LINGO1 and RPN3A in KI/del)

Under standard conditions, 713 genes were significantly altered in KI/KI cells and 787 in KI/del cells compared to NTC (adjusted *p*-value < 0.05). Notably, while these numbers suggest substantial transcriptional dysregulation, ZNF536 itself was not identified among differentially expressed genes (DEGs). This apparent paradox is explained by ZNF56's complex expression pattern involving 12 different isoforms, which likely masks detection of significant changes in total transcript levels. This finding underscores the importance of considering transcript complexity when interpreting knockout effects.

The overlap analysis revealed an asymmetric pattern of gene regulation: 225 downregulated genes (17.8%) were shared between mutant lines, compared to only 105 upregulated genes (8.7%). This bias toward conserved downregulation suggests that ZNF536 may primarily function as a transcriptional activator in these cells (Figure 7).

### GO enrichment analysis reveals distinct functional impact of ZNF536 variants

To systematically characterize how ZNF536-loss perturbs transcriptional programs under basal and differentiation-dependent conditions, we performed pathway enrichment analyses on RNA-seq data from NTC, KI/KI, and KI/del lines. This approach prioritized neurodevelopmental trajectories implicated in SZ schizophrenia pathogenesis. Thus, gene ontology (GO) enrichment analysis showed that different ZNF536 variants affect gene regulation in unique ways, hinting at varied causes of disease. In cells where ZNF536 is completely non-functional (KI/KI), there's a notable drop in the activity of genes tied to chromatin organization. This includes lower expression of nucleosome assembly genes like HIST1H2BK, HIST1H2AC, and HIST2H2BE, as well as chromatin remodeling genes such as SMARCA2 and CHD2. These changes suggest that ZNF536 is essential for keeping chromatin—the structure that packages DNA—properly organized. Moreover, these cells struggle with neuronal development. In turn, this might explain the delayed neuronal differentiation of both mutants upon ATRE treatment. Genes critical for axonogenesis (the growth of nerve fibers), including NEFL, GAP43, and DPYSL2, are less active. Similarly, genes involved in neuronal projection development, like DCX and BASP1, show reduced expression. This points to ZNF536's important role in supporting healthy brain cell growth (Figure 8).

In contrast, cells carrying the regulatory region deletion (KI/del) displayed a markedly different pattern focused on synaptic function. We observed coordinated downregulation of neurotransmitter secretion genes SYT1, SNAP25, and SYN1, accompanied by reduced expression of synapse organization factors SHANK2, DLG2, and NRXN2. Chemical synaptic transmission genes GRIA3 and GRIK1 also showed significant reduction. This pattern of synaptic gene suppression was coupled with broader downregulation of nervous system development pathways.

### Enrichment analyses by KEGG pathways, and disease ontology gene sets

**Figure 9:**
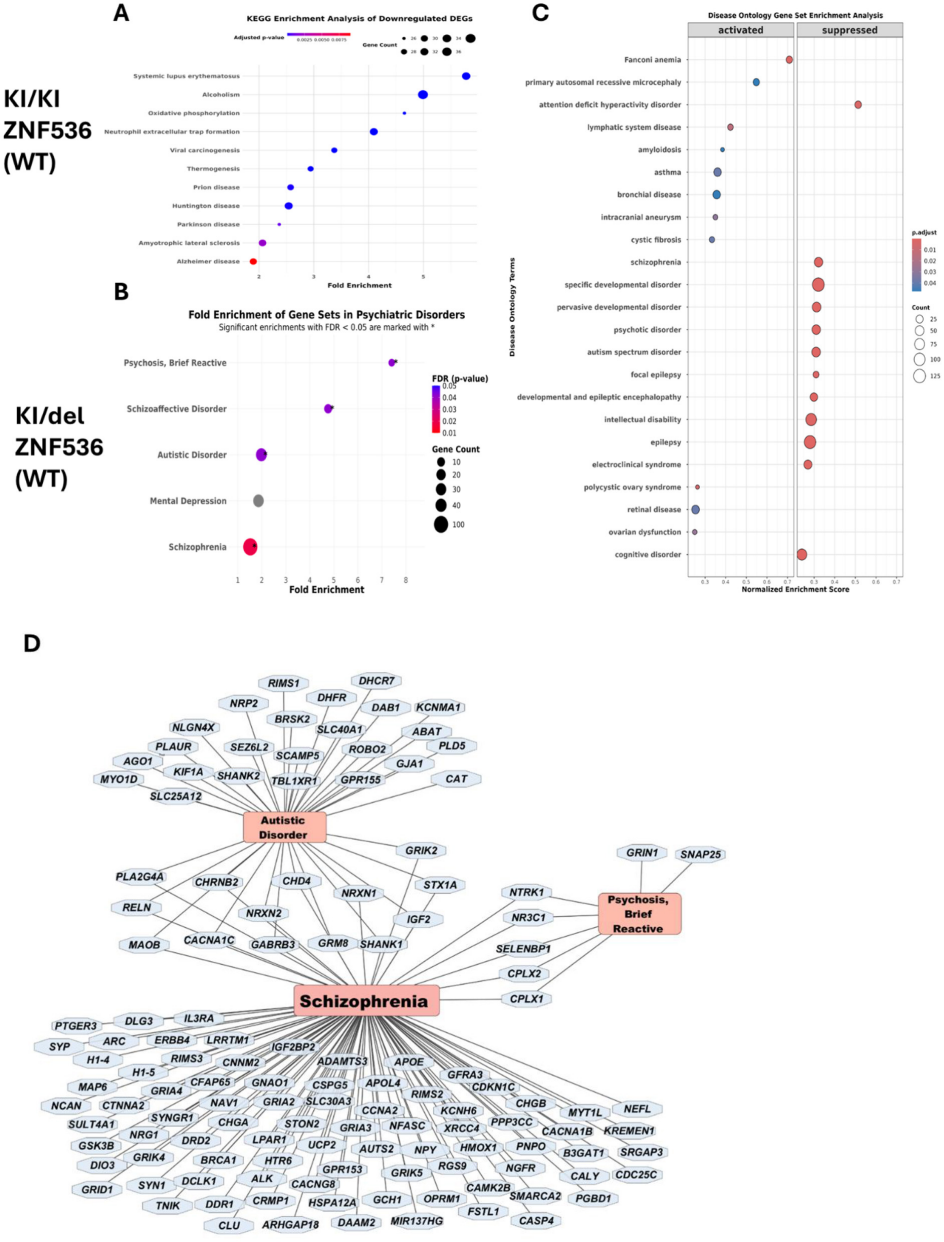
ZNF536 dysfunction in undifferentiated cells is linked to SZ pathogenesis and neurodegeneration. Enrichment for the disease-associated genes among DEGs based on KEGG for KI/KI cell line **(A)**, for KI/del cell line according to the DisGeNET **(B)** and to DOSE databases **(C)**. Gene-disease association network for KI/del cell line **(D)**.

To elucidate the biological mechanisms affected by ZNF536 KI with frameshift, we performed pathway enrichment analyses using DisGeNET, KEGG pathways, and Disease Ontology Gene Sets. The two mutant lines (KI/KI and KI/del) exhibited markedly different enrichment patterns, suggesting distinct pathogenic mechanisms. In the KI/KI line, KEGG pathway analysis revealed significant enrichment in neurodegenerative pathways, particularly Alzheimer's disease (2.1-fold, *p* < 0.01) and amyotrophic lateral sclerosis (2.3-fold, *p* < 0.05). These cells also demonstrated strong enrichment in oxidative phosphorylation pathways (3.8-fold, *p* < 0.05), indicating primary effects on cellular metabolism. Notably, DisGeNET analysis revealed no significant enrichment for psychiatric disorders in this line. In contrast, the KI/del line demonstrated strong associations with psychiatric and neurodevelopmental disorders. DisGeNET analysis identified significant enrichment for SZ (1.8-fold, FDR < 0.05), schizoaffective disorder (4.8-fold, FDR < 0.05), and autistic disorder (2.1-fold, FDR < 0.05). We also depicted on disease-gene network analysis revealed genes including key synaptic proteins (CPLX1, CPLX2, SYN1) and neurodevelopmental regulators (GRIA3, GRIK1; Figure 9).

These distinct enrichment patterns suggest that the genetic region of ZNF536 deleted in KI/del cells contains regulatory elements crucial for psychiatric disease-related gene expression. The disease-gene network analysis (Figure 9) supported this hypothesis, revealing substantial overlap between differentially expressed genes in KI/del cells and known psychiatric disorder-associated genes.

Collectively, these findings demonstrate that inactivation of ZNF536 via KI with frameshift primarily affects cellular metabolism and neurodegenerative pathways, while deletion of the ZNF536's region in compound heterozygous sample specifically disrupts psychiatric disorder-related gene networks. Therefore, we speculate that ZNF56's role in psychiatric disorders is mediated predominantly through its regulatory elements in its gene structure rather than its protein-coding function.

### Gene set enrichment analysis in the KI/del line

**Figure 10:**
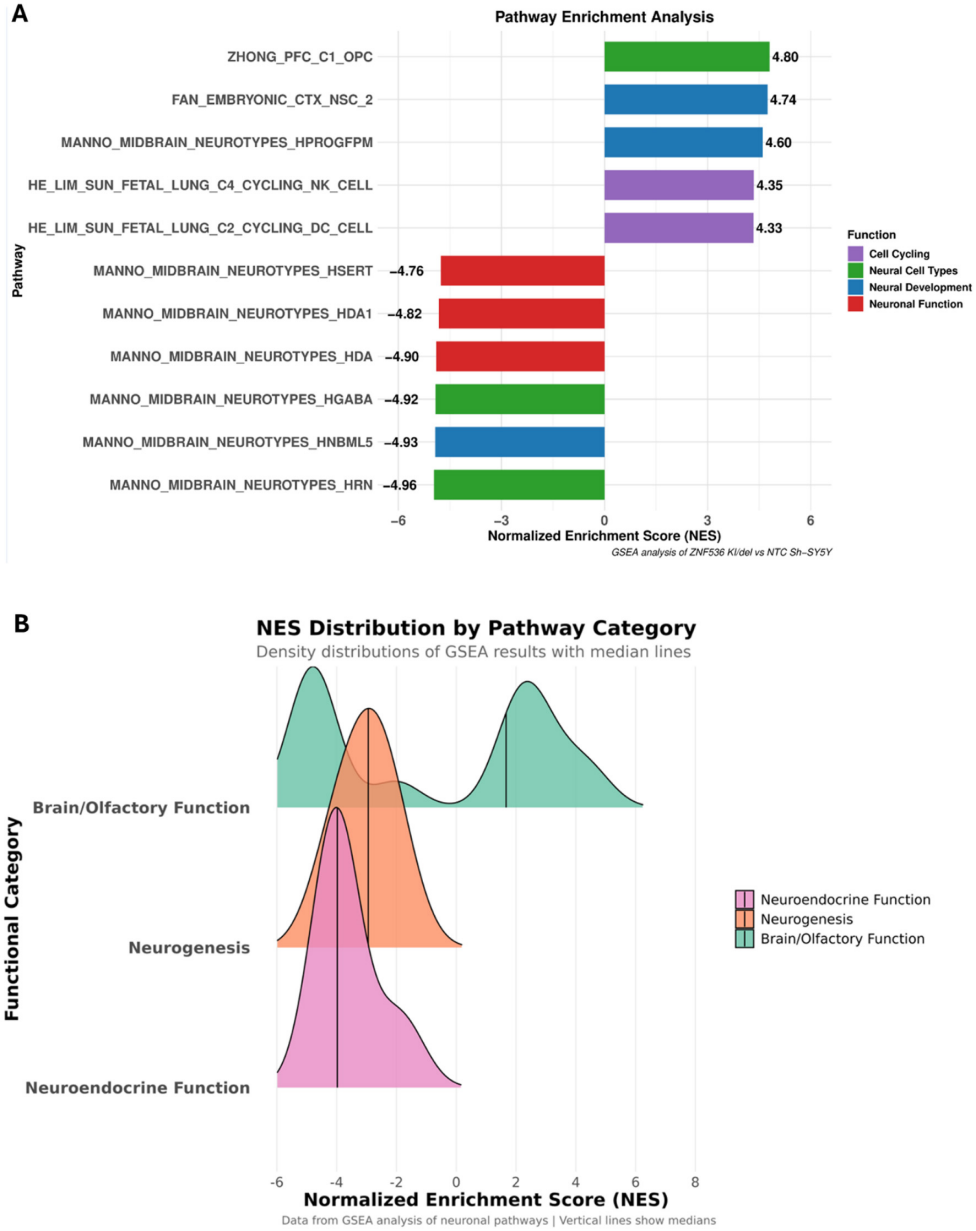
This is a figure. Schemes follow the same formatting. Pathway enrichment analysis (bar plot): bar plot illustrates the top enriched gene sets identified by GSEA in ZNF536 KI/del vs. control SH-SY5Y cells. Each bar represents a pathway, with the Normalized Enrichment Score (NES) shown on the *x*-axis **(A)**. NES distribution by pathway category (density plot) density plot shows the distribution of NES values for three major functional categories: Neuroendocrine Function (pink), Neurogenesis (orange), and Brain/Olfactory Function (green) **(B)**. Density Curves: Each curve represents the distribution of NES values within a functional group. Median Lines: Vertical lines denote the median NES for each category.

To systematically characterize the biological pathways altered by ZNF536 KI with frameshift, we performed GSEA on differentially expressed genes (DEGs) from the KI/del (compound heterozygous) line using the Molecular Signatures Database (v2023.1.Hs). We focused on neuronal and developmental gene sets, given ZNF536's putative role in neurodevelopment (Figure 10). Multiple Manno midbrain neurotype sets—particularly MANNO_MIDBRAIN_NEUROTYPES_HDA and MANNO_MIDBRAIN_NEUROTYPES_HRN—displayed strong negative enrichment (NES < −4.0), indicating widespread downregulation of genes vital for neuronal differentiation and function. Among the most significantly affected genes were NRXN1/2 (neurexins), which are essential for synaptic adhesion and transmission (Lin et al., 2023); SNAP25, which is crucial for synaptic vesicle docking and exocytosis (Antonucci et al., 2016); SYP (synaptophysin), involved in synaptic vesicle trafficking (Kwon and Chapman, 2011); DCX (doublecortin), which regulates neuronal migration and microtubule stabilization (Sébastien et al., 2025); and MAP2, which is key to dendritic structure and function (DeGiosio et al., 2022). We also observed negative enrichment in pathways related to neuroendocrine development, such as DESCARTES_FETAL_INTESTINE_ENS_NEURONS (NES = −4.32). Conversely, early progenitor-associated gene sets (e.g., MANNO_MIDBRAIN_NEUROTYPES_HPROGFPM) showed positive enrichment (NES > 3.0), suggesting an upregulation of genes typically active in undifferentiated neural precursors. This bimodal distribution—suppression of mature neuronal markers alongside increased progenitor programs—implies that ZNF536 acts as both an activator and repressor, guiding cells from early developmental states toward terminal differentiation. In total, the downregulated neurotype signatures contained an overlapping cluster of 53 genes. Several appeared in multiple sets, underscoring their broad importance in neuronal processes. These included BASP1, which influences growth cone morphology and axonal development (Mac Donald and Iulianella, 2022); ATP1A3, which maintains ion gradients crucial for neuronal excitability (Smith et al., 2021); NRXN1/2, which are key for synapse formation (Lin et al., 2023); and SNAP25, which mediates synaptic vesicle fusion (Antonucci et al., 2016).

These findings corroborate earlier observations of impaired neuronal maturation in ZNF536 mutant cells, highlighting those critical processes—such as synaptogenesis, cytoskeletal reorganization, and neuronal subtype specification—are particularly sensitive to ZNF536 inactivation. Along with results from the KI/KI model, these GSEA outcomes underscore ZNF536's pleiotropic influence on neural development, where LoF disrupts both synaptic integrity and the transition from progenitor to differentiated neuron.

### ZNF536 modulates retinoic acid responsiveness and neuronal differentiation programs

**Figure 11:**
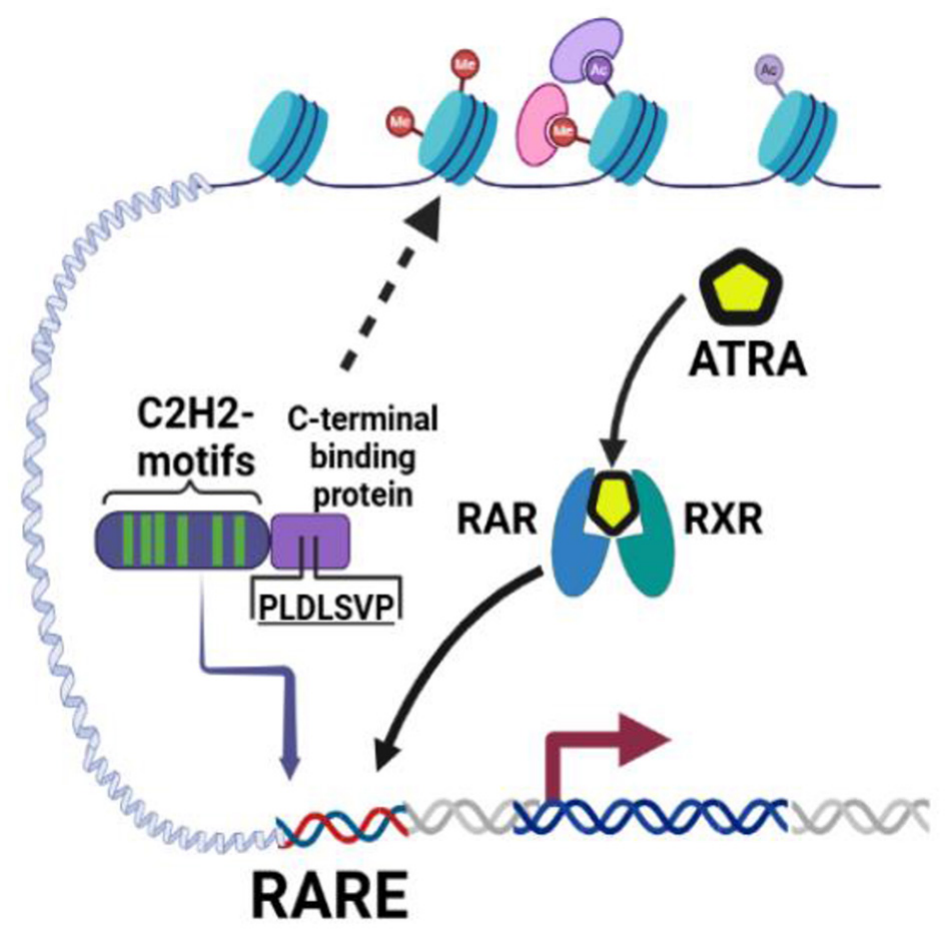
Retinoic acid (ATRA) RA binds to the retinoic acid RA receptor (RAR), which heterodimerizes with the retinoid X receptor (RXR) to activate gene transcription at RAREs (Qin et al., 2009). In parallel, ZNF536, equipped with 10 C2H2-type zinc finger motifs (green), can bind RAREs (or nearby regulatory DNA regions) and potentially compete with RAR/RXR for RARE occupancy. By recruiting C-terminal binding protein (CtBP) through its conserved PLDLSVP motif, ZNF536 assembles co-repressor complexes containing histone-modifying enzymes (e.g., histone deacetylases, lysine demethylases). These complexes condense chromatin (as indicated by altered histone marks in purple and pink), thereby inhibiting transcription of key neurogenic genes.

Previous work has shown that ZNF536 competes with RARs for binding at retinoic acid response elements (RAREs, DR1-DR5; Figure 11). To examine this regulatory relationship in detail, we compared the global transcriptomic responses of NTC cell line, homozygous knock-in of ZNF536 with frameshift (KI/KI) and compound heterozygous (KI/del) cells under basal conditions and following ATRA treatment.

As expected, NTC cells exhibited significant induction of canonical RA targets upon ATRA exposure. Known RAR-regulated genes (e.g., *CYP26B1, CRABP2, CYP26A1, RARB*) were markedly upregulated (log_2_FC > 4, *p* < 0.001), reinforcing the well-characterized RA-driven differentiation pathway in SH-SY5Y cells. In contrast, KI/KI cells displayed a severely diminished ATRA response. Notably, *RARB* expression was dramatically reduced (log_2_FC = 0.9 vs. 3.9 in NTC), and several neuronal markers (*GAP43, NTRK1, NGFR*) showed altered regulation. In turn, the KI/del line demonstrated a partial ATRA response, with *RARB* expression higher than in KI/KI but still lower than in NTC cells.

Interestingly, *ASCL1* was upregulated under both basal and ATRA-treated conditions suggesting that the large deletion of ZNF536 genetic region removed regulatory elements that normally repress *ASCL1* independently of RA. To investigate this hypothesis, we performed motif analysis of the deleted genetic region of ZNF536 in KI/del sample using MoLoTool (Loudig et al., 2005), which identified three high-confidence ASCL1 binding sites with significant *p*-values (*p* = 4.508e-5, *p* = 5.834e-5, and *p* = 1.227e-5). These motifs conform to the canonical E-box sequence (CAGCTG) and are located at positions: chr19: 30450251–30450260 (–); chr19:30448935–30448946 (+) and chr19:30539754–30539765 (+). The presence of conserved binding sites specifically within this region, combined with the selective upregulation of *ASCL1* in KI/del but not KI/KI cells, suggests that ZNF56's deleted region contains functional regulatory elements that modulate *ASCL1* expression through a feedback mechanism.

Thus, in addition to mediating RA sensitivity, *ZNF536* appears to harbor discrete cis-regulatory domains that modulate key neurogenic factors outside the classical RAR signaling axis.

### ZNF536 exhibits distinct regulation of RARE-containing genes

**Figure 12:**
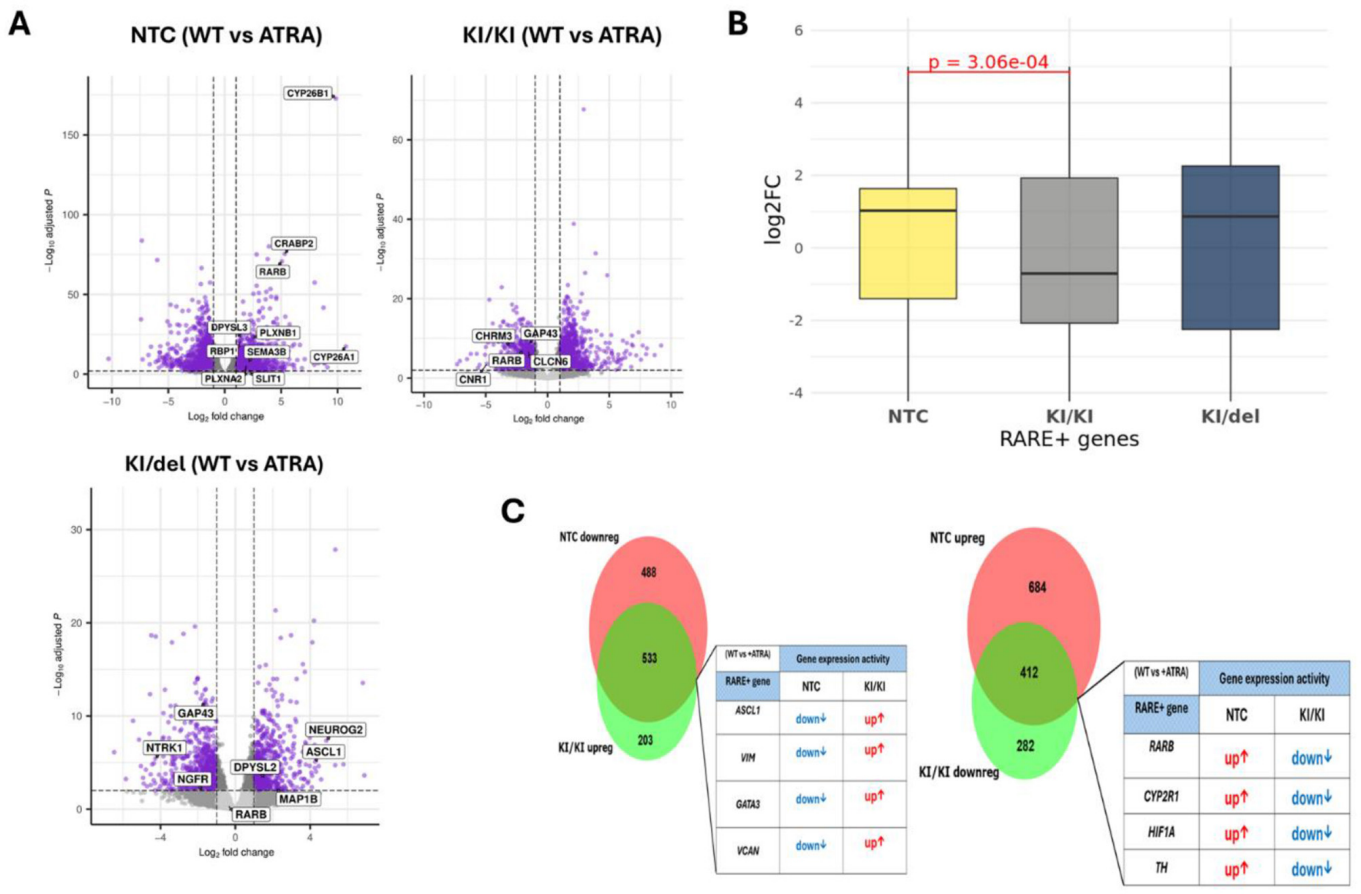
Schemes follow the same formatting. ZNF536 modulates RA-responsive gene expression and exhibits inverse transcriptional regulation. Volcano plots showing differential gene expression profiles in response to ATRA treatment (10 μm, 10 days) in NTC (top left), KI/KI (top right), and KI/del **(bottom)** cells. Gray dots represent genes below significance threshold; purple dots indicate significantly changed genes (adjusted *p*-value < 0.05, |log2FC| > 1). Selected genes are labeled. *X*-axis shows log2 fold change; *Y*-axis shows –log10(adjusted *p*-value) **(A)**. Distribution of log2 fold changes for RARE+ genes across genotypes following ATRA treatment. Box plots show median (horizontal line), interquartile range (box), and range (whiskers). Significant difference between conditions was observed (*p* = 3.06e-04), indicating altered RA responsiveness in KI/KI cell line in comparison with NTC and KI/del **(B)**. Venn diagrams depicting overlap between differentially expressed RARE+ genes. RARE+ genes showed reciprocal regulation between NTC and KI/KI conditions: genes typically downregulated in control cells became activated in ZNF536-deficient cells (533 genes, 11.09-fold enrichment) and vice versa (412 genes, 8.47-fold enrichment). This inverse pattern suggests ZNF536 normally maintains proper directionality of RA-responsive gene expression by altered histone marks in purple and pink, thereby inhibiting transcription of key neurogenic genes **(C)**.

To further elucidate how ZNF536 modulates RA-dependent transcription, we examined the distribution of log_2_ fold changes (log_2_FC) for genes harboring RAREs within their promoters. Previous studies have established that ZNF536 functions as a transcriptional regulator that competes with RARs for binding at RAREs. RAREs are specific DNA sequences, typically consisting of direct repeats separated by 1–5 nucleotides (DR1–DR5) that serve as binding sites for RAR/RXR heterodimers to activate gene transcription in response to RA (Loudig et al., 2005; Figure 12A).

This analysis revealed a significant difference between KI/KI and NTC cells (*p* = 3.06 × 10^−4^), with KI/KI exhibiting an overall reduction in RARE^+^ gene expression (Figure 6). These genome-wide results align with our prior qPCR findings: RARB expression was specifically downregulated in KI/KI compared to both NTC and KI/del lines. To investigate whether KO via KI of ZNF536 reverses normal RA-driven transcriptional programs, we analyzed the overlap between RARE^+^ genes differentially expressed in NTC and KI/KI conditions. We observed significant bidirectional inverse correlation in gene regulation. Among RARE^+^ genes upregulated in NTC, 412 were downregulated in KI/KI (8.47-fold enrichment over random expectation, *p* < 1e-300, hypergeometric test). Conversely, of RARE^+^ genes downregulated in NTC, 533 were upregulated in KI/KI (11.09-fold enrichment, *p* < 1e-10). This pronounced reciprocal regulation, particularly strong among RARE^+^ genes compared to the total transcriptome (11.09x vs. 7.57x enrichment), suggests that ZNF536 specifically modulates RA-responsive gene expression (Figure 12C).

Such reciprocal regulation suggests that ZNF536 functions as both an activator and repressor, maintaining the balance of RA-responsive and neuronal identity genes. The enrichment of RARE^+^ genes among inversely regulated transcripts indicates a direct role in modulating retinoic acid response elements—potentially via interactions with the RA receptor complex or associated cofactors. Disfunction of ZNF536 in KI/KI cells thus triggers broad transcriptional dysregulation, notably impacting genes tied to RA signaling and neuronal differentiation.

### ZNF536 orchestrates RA-responsive and allele-specific transcriptional networks

**Figure 13:**
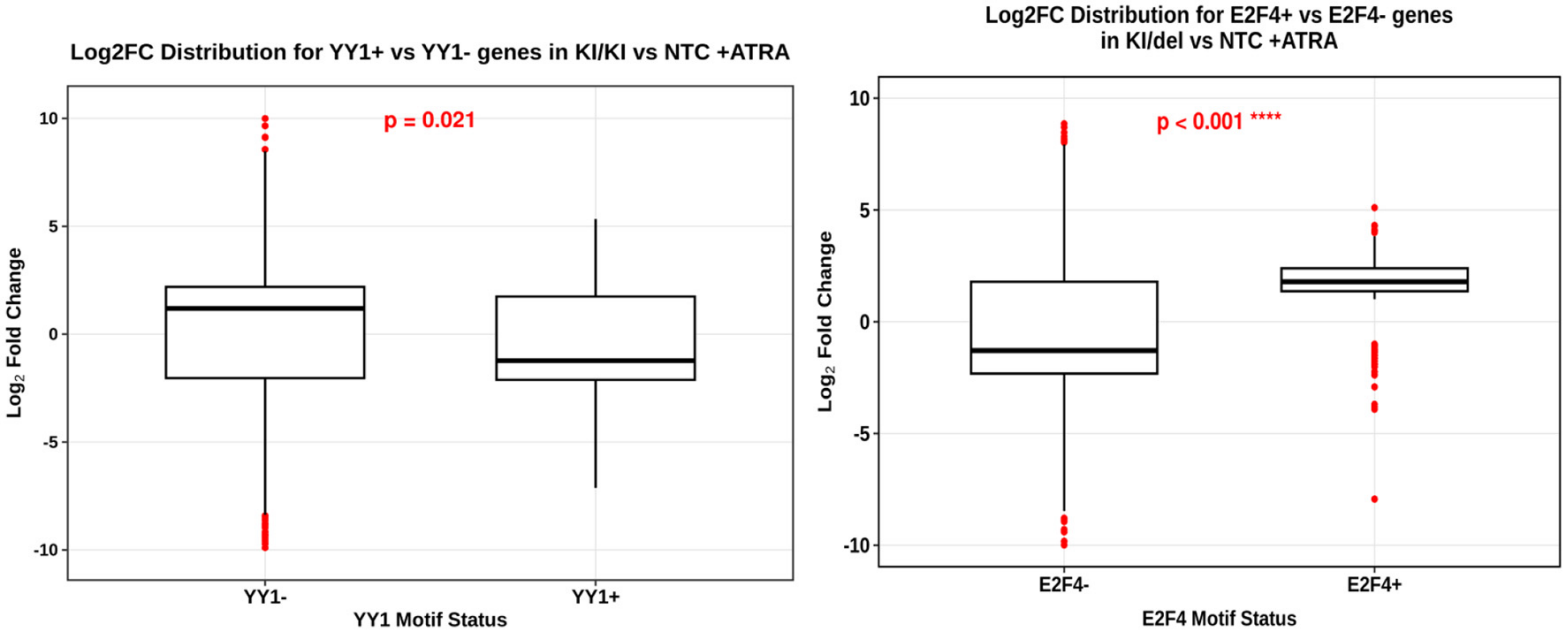
Transcription Factor Motif Analysis Reveals Divergent Regulatory Programs in ZNF536 Mutant Lines. In KI/KI cells under ATRA treatment, genes harboring YY1 motifs (YY1^+^) display higher log_2_FC compared to genes lacking YY1 sites (YY1^−^), suggesting YY1-dependent regulation (*p* = 0.021).

Upon confirming the function of ZNF536 in the regulation of RARE^+^ gene expression, our objective was to delineate the transcription factor (TF) networks that are differentially activated in ZNF536 mutant lines (KI/KI and KI/del) throughout ATRA-induced differentiation. To systematically delineate the transcription factor pathways disrupted by ZNF536 mutations, we assessed the enrichment of transcription factors among differentially expressed genes (DEGs), comparing the control NTC with the mutant lines subjected to ATRA treatment, utilizing existing ChIP-seq data provided by the ENCODE consortium. By synthesizing these binding profiles with HOMER motif analysis and the observed patterns of differential gene expression, we identified unique compensatory regulatory programs corresponding to each mutant allele (Figure 13).

ENCODE ChIP-seq screening of DEGs in KI/KI mutants revealed significant enrichment for YY1 (Yin Yang 1) binding sites (*p* = 2.07 × 10^−12^; FDR = 3.8 × 10^−11^; OR = 1.532), with specificity to SK-N-SH neuroblastoma cells. HOMER motif analysis corroborated this, identifying a conserved YY1 motif (YCARGATGGCGG, *p* = 1 × 10^−15^) enriched in promoters of DEGs. Of 1,915 DEGs, 486 harbored YY1 motifs (YY1^+^), while 1,429 lacked these sites (YY1^−^). Though YY1^+^ genes showed only modest expression shifts (Wilcoxon *p* = 0.021), their bidirectional regulation—mirroring ZNF536's dual role in RA signaling—suggests YY1 compensates for complete ZNF536 loss by fine-tuning neuronal and RA-responsive genes. This cell-type–specific enrichment aligns with YY1's known role in stabilizing neurodevelopmental programs under genetic stress.

In KI/del cells, ENCODE ChIP-seq analysis of DEGs identified E2F4 (E2F transcription factor 4) as the dominant enriched TF (*p* = 7.49 × 10^−27^; FDR = 4.14 × 10^−24^; OR = 15.62), with 273 of 2,105 DEGs harboring E2F4 motifs (E2F4^+^). Surprisingly, E2F4^+^ genes exhibited strong positive regulation (median log_2_FC > 0), whereas E2F4^−^ genes trended downward (median log_2_FC < 0; Wilcoxon *p* < 0.001). This bifurcation suggests partial ZNF536 disruption (KI/del) permits residual protein function to interact with E2F4, redirecting ATRA-induced differentiation toward cell cycle or alternative neurogenic pathways. The 15.62-fold enrichment of E2F4 binding sites underscores its centrality to this allele-specific response.

By contrast, in KI/del cells, genes containing E2F4 motifs (E2F4^+^) exhibit a significantly different expression distribution from E2F4^−^ genes (*p* < 0.001), with E2F4^+^ genes generally showing greater induction under ATRA. Overall, these results reveal how distinct ZNF536 mutations engage alternative compensatory pathways to rewire transcription. In KI/KI cells, complete ZNF536 loss confers a modest yet noticeable dependence on YY1 (OR = 1.532), aligning with the gradual dysregulation observed in RARE^+^ genes. Conversely, KI/del cells exhibit a more pronounced shift toward E2F4-driven programs, exploiting partial ZNF536 function to “hijack” transcriptional outputs.

## Discussion

Using CRISPR knockouts in SH-SY5Y neuroblastoma cells, we mapped allele-specific effects of loss of ZNF536 on retinoic acid–induced neuronal differentiation. Biallelic KI/KI clones showed a marked failure of neuritogenesis, whereas KI/del clones displayed an intermediate impairment. ranscriptomics defined two distinguishable axes: KI/KI downregulated chromatin-organization programs (nucleosome assembly; remodelers), with KI/del tending to downregulate synaptic/neurodevelopmental networks with downregulation of TSHZ3 (Figures 3, 4, 6, 8, 9). Conceptually, this is consistent with the TOP2B knockout phenotype that also interferes with RA-induced differentiation in SH-SY5Y (Khazeem et al., 2022). Our novel aspect is testing ATRA + BDNF, which partially rescues neurite outgrowth in cells lacking ZNF536—something not addressed in the TOP2B paper—implicating rescue signaling competent to usurp parts of ZNF536-dependent transcription (Figures 3, 4). Mechanistically, KI/KI cells exhibited reduced expression of histone genes and remodelers (e.g., HIST1H2BK/H2AC/H2BE; SMARCA2; CHD2), consistent with a “chromatin-delay” model in which RA fails to timely engage long neurodevelopmental loci. Moderate YY1 target enrichment indicates limited compensation that is unable to restore a complete differentiation program (Figures 8A, 13). In KI/del, protein-coding loss is separated from intronic enhancer disruption by genotype with TSHZ3 downregulation coupled with a synaptic signature (CPLX1/2, SYN1, GRIA3, GRIK1). Extensive E2F4 motif enrichment among ATRA-responsive DEGs (~15.6-fold) suggests cell-cycle–linked rewiring instead of canonical RA programs restoration (Figures 6, 9, 13).

In KI/del, the genotype separates protein-coding loss from intronic enhancer disruption, with TSHZ3 downregulation and a synaptic signature (CPLX1/2, SYN1, GRIA3, GRIK1). Strong E2F4 motif enrichment among ATRA-responsive DEGs (~15.6-fold) indicates cell-cycle–linked rewiring rather than restoration of canonical RA programs (Figures 6, 9, 13). Together, the allelic contrasts clarify how coding vs. non-coding ZNF536 variation may differentially route risk through chromatin and synaptic pathways.

The ATRA + BDNF rescue likely reflects one or both of the following testable hypotheses: (1) activation of TrkB–BDNF–PI3K signaling (permitted by RA-induced TrkB) that promotes neurite extension and survival; and/or (2) engagement of a NEUROG2-centered parallel program that circumvents ZNF536-dependent steps (Kaplan et al., 1993; Nishida et al., 2008; Vainorius et al., 2023).

Discrepancies seen with experiments on stem-like systems (e.g., HEK293T/P19 from Qin et al.) most likely represent cell-state–dependent products of RA–ZNF536 signaling. Although ZNF536 might function as a safeguard against early differentiation in stem-like contexts, our data indicate that in an even more committed state such as neuroblastoma ZNF536 activity is additionally relevant to ordered programs of RA. Thus, cell-state dependency helps to explain why our deficiency here causes—rather than corrects—maturation to a neuronal phenotype.

Consequently, several constraints shape the interpretation of these findings. SH-SY5Y is a transformed line with limited capacity to model primary neuronal development, and our biallelic knockouts—though mechanistically informative—cannot capture the heterozygous dosage effects likely relevant to disease. Despite these limitations, our work establishes critical mechanistic anchors for more physiological studies.

Future work should leverage isogenic iPSC-derived cortical neurons harboring heterozygous ZNF536 mutations that mirror patient genotypes. *De novo* mutations in ZNF536 exon 2 have been identified in ASD probands, establishing haploinsufficiency as clinically relevant. These models would address critical gaps: (1) how ASD-associated ZNF536 mutations affect RA responsiveness and neuronal maturation in dose-sensitive contexts, (2) whether the intronic enhancers (GH19J030448, hs384.0) independently regulate TSHZ3 and synaptic networks, and (3) which specific regulatory elements within the 103-kb deleted region contribute to neurodevelopmental phenotypes.

## Data Availability

The raw RNA-sequencing data supporting the conclusions of this article have been deposited in NCBI's Gene Expression Omnibus (GEO) under accession number GSE304368 (“RNA-seq of CRISPR/Cas9-Mediated ZNF536 Knockout in SH-SY5Y Cells Undergoing ATRA-Induced Differentiation”) and are publicly accessible at: https://www.ncbi.nlm.nih.gov/geo/query/acc.cgi?acc=GSE304368.
