# Supplementary figures and images for: Schizophrenia risk gene ZNF536 modulates retinoic acid response and neuronal gene networks in SH-SY5Y cells
Source: Front Mol Neurosci. 2025 Oct 14;18:1671354. doi: 10.3389/fnmol.2025.1671354 (PMC12558893; doi:10.3389/fnmol.2025.1671354)

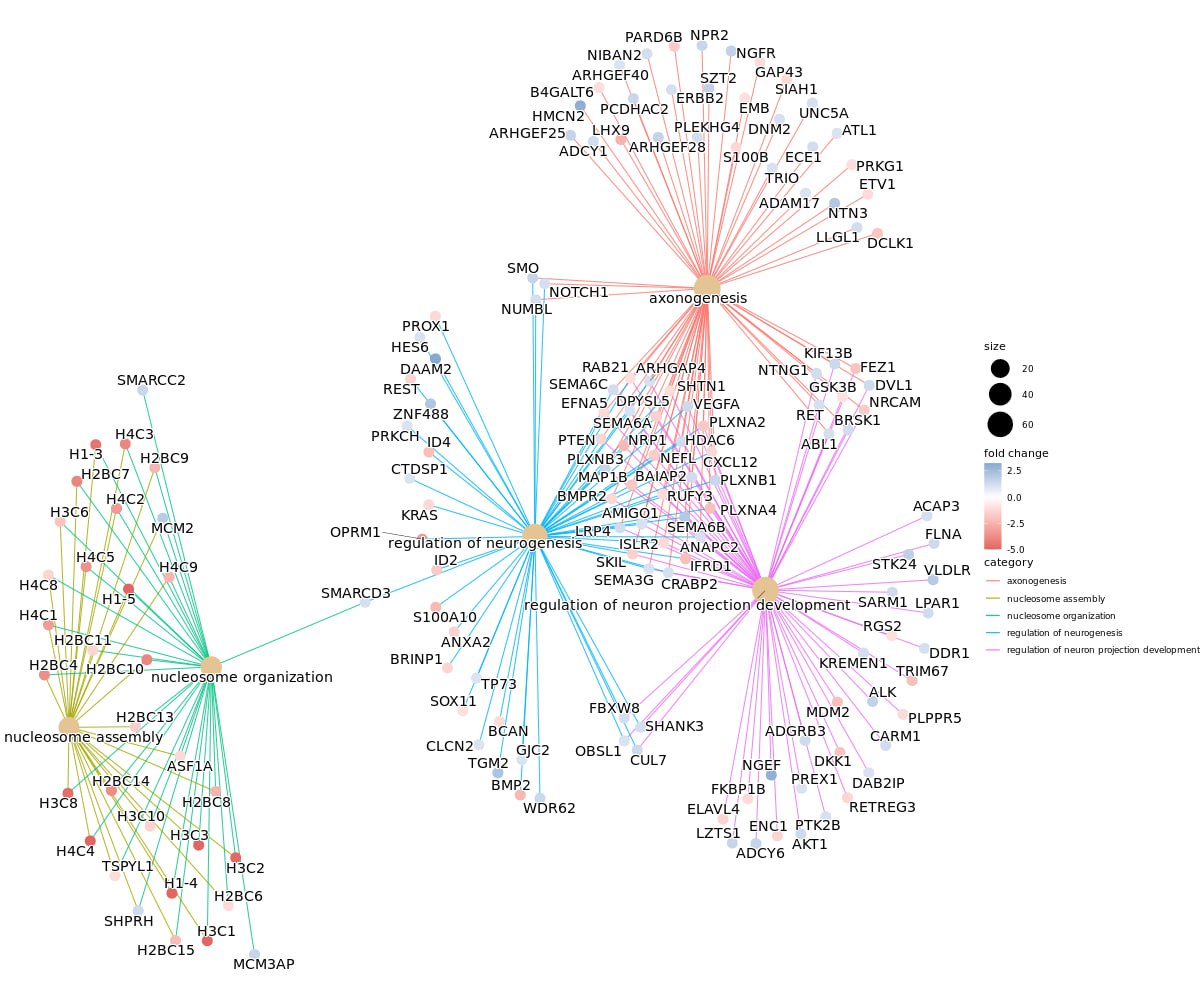

Supplement: Supplementary file 1 [file Data_Sheet_1.zip › Fig_S1_KI-KI_cnetplot.jpg]

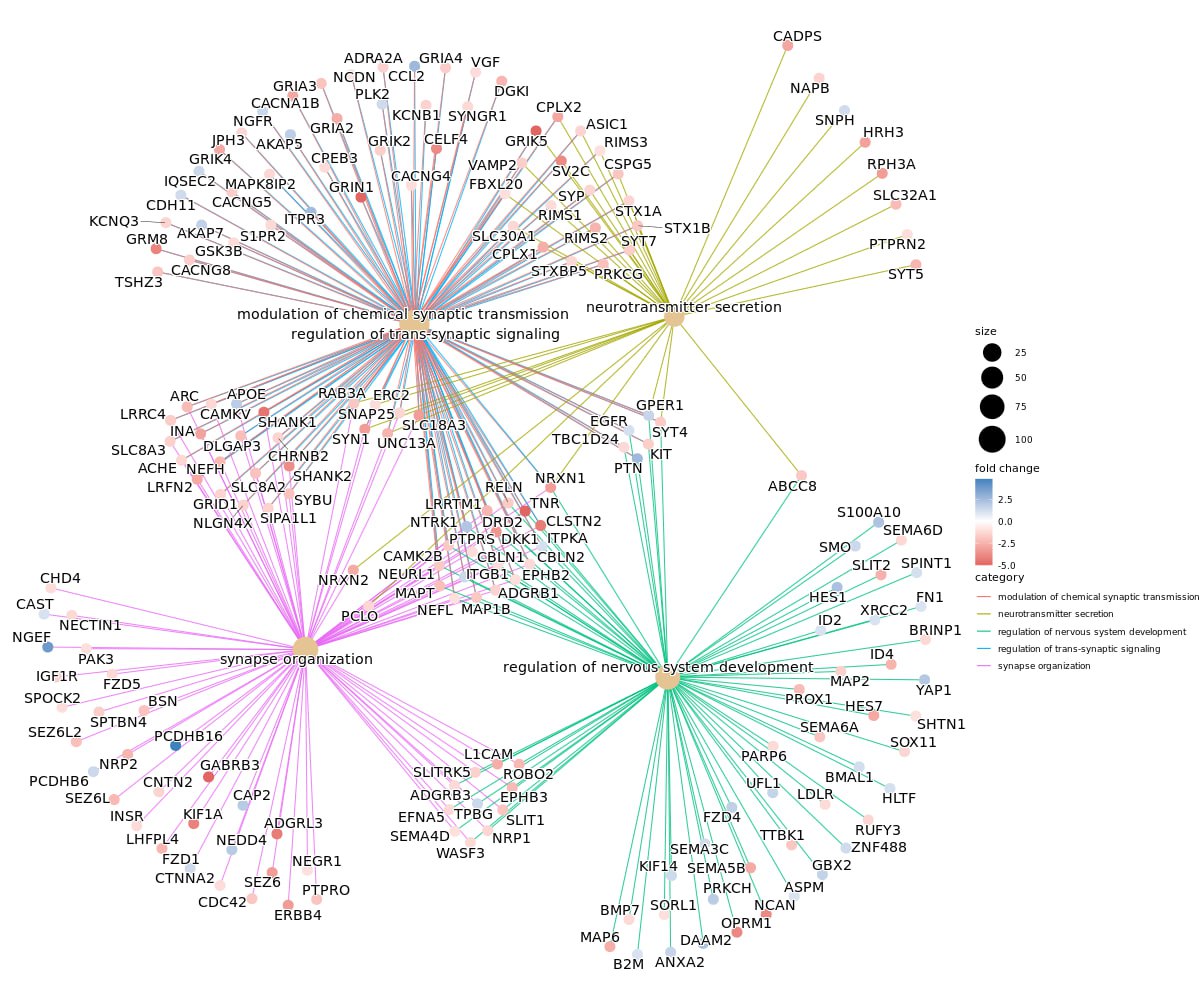

Supplement: Supplementary file 1 [file Data_Sheet_1.zip › Fig_S2_KI_del_cnetplot.jpg]

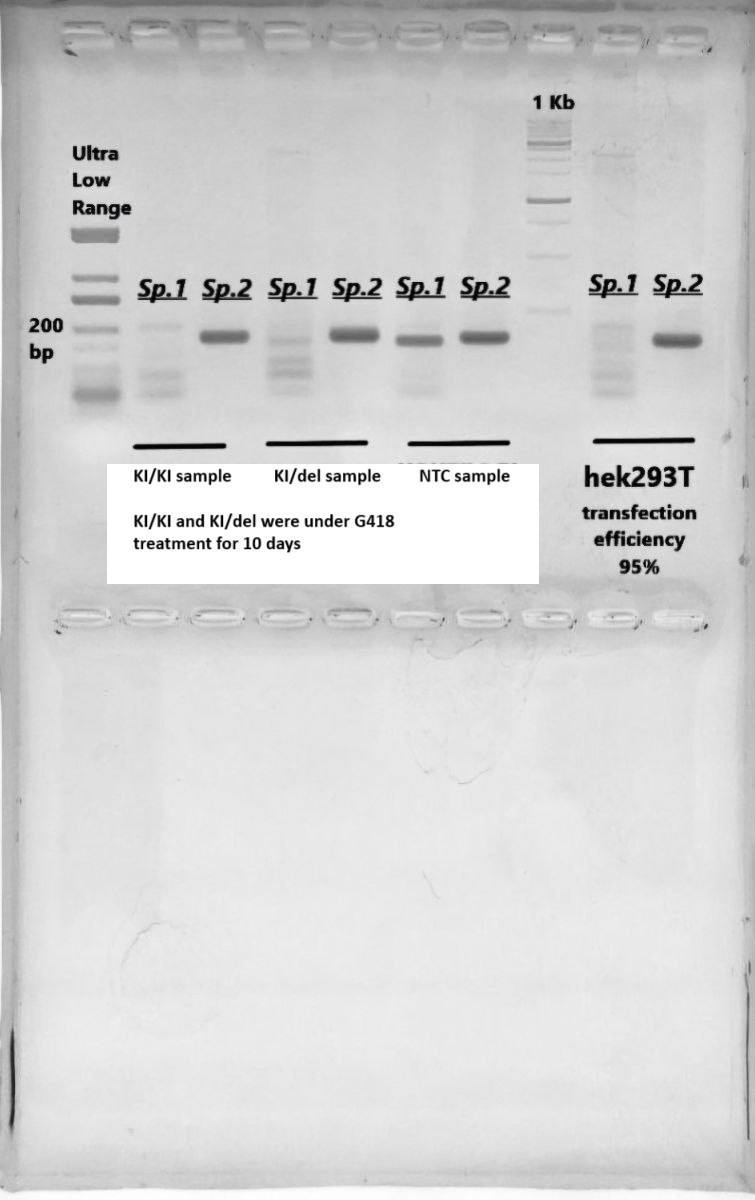

Supplement: Supplementary file 1 [file Data_Sheet_1.zip › Fig_S3_check_pcr_full_knockin_ZNF536.tif]

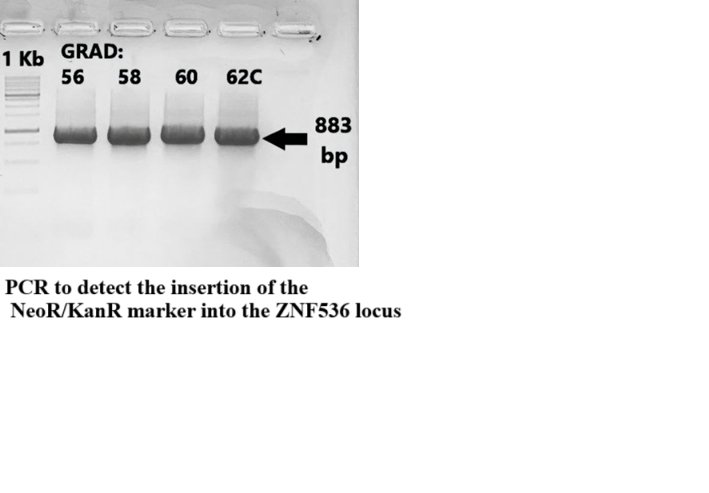

Supplement: Supplementary file 1 [file Data_Sheet_1.zip › Fig_S4_knockin_insertion_pcr.tif]

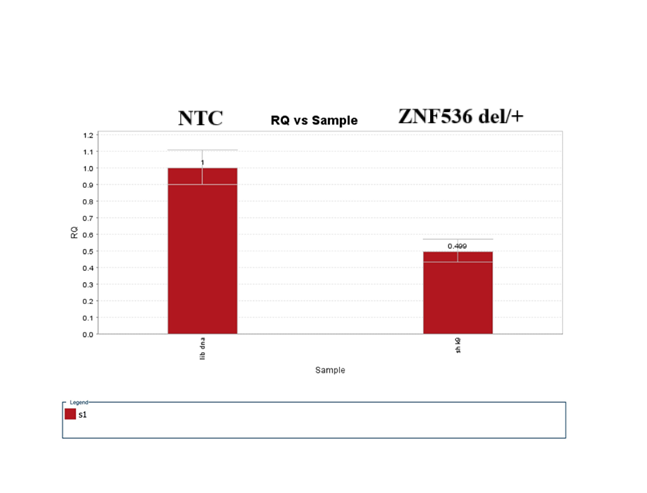

Supplement: Supplementary file 1 [file Data_Sheet_1.zip › Fig_S5_ZNF536_heterozygous_deletion_qPCR.tif]
